# Identification of MsHsp20 Gene Family in *Malus sieversii* and Functional Characterization of MsHsp16.9 in Heat Tolerance

**DOI:** 10.3389/fpls.2017.01761

**Published:** 2017-11-01

**Authors:** Meiling Yang, Yunxiu Zhang, Huanhuan Zhang, Hongbin Wang, Tao Wei, Shiyou Che, Lipeng Zhang, Baoquan Hu, Hong Long, Wenqin Song, Weiwei Yu, Guorong Yan

**Affiliations:** ^1^Department of Genetics, College of Life Sciences, Nankai University, Tianjin, China; ^2^Department of Pomology, College of Horticulture and Landscape, Tianjin Agricultural University, Tianjin, China

**Keywords:** *Malus sieversii*, RNA-Seq, *MsHsp20* family, *MsHsp16.9*, expression profile, heat stress

## Abstract

Heat shock proteins (Hsps) are common molecular chaperones present in all plants that accumulate in response to abiotic stress. Small heat shock proteins (sHsps) play important roles in alleviating diverse abiotic stresses, especially heat stress. However, very little is known about the *MsHsp20* gene family in the wild apple *Malus sieversii*, a precious germplasm resource with excellent resistance characteristics. In this study, 12 putative *M. sieversii Hsp20* genes were identified from RNA-Seq data and analyzed in terms of gene structure and phylogenetic relationships. A new Hsp20 gene, *MsHsp16.9*, was cloned and its function studied in response to stress. *MsHsp16.9* expression was strongly induced by heat, and transgenic *Arabidopsis* plants overexpressing *MsHsp16.9* displayed improved heat resistance, enhanced antioxidant enzyme activity, and decreased peroxide content. Overexpression of *MsHsp16.9* did not alter the growth or development under normal conditions, or the hypersensitivity to exogenous ABA. Gene expression analysis indicated that *MsHsp16.9* mainly modulates the expression of proteins involved in antioxidant enzyme synthesis, as well as ABA-independent stress signaling in 35S:*MsHsp16.9*-L11. However, *MsHsp16.9* could activate ABA-dependent signaling pathways in all transgenic plants. Additionally, *MsHsp16.9* may function alongside *AtHsp70* to maintain protein homeostasis and protect against cell damage. Our results suggest that *MsHsp16.9* is a protein chaperone that positively regulates antioxidant enzyme activity and ABA-dependent and independent signaling pathway to attenuate plant responses to severe stress. Transgenic plants exhibited luxuriant growth in high temperature environments.

## Introduction

Extreme environments can induce complex biotic and abiotic stress in plants (Cramer et al., 2011). Among the numerous environmental factors, increased global warming is likely to seriously affect the growth and development of plants (Yu et al., 2012). Thermal stress disturbs cell homeostasis and disrupts growth and development, and can lead to death (Kotak et al., 2007). As sessile organisms, plants have evolved a complex set of responses to deal with heat stresses. Transcriptome analysis of *Arabidopsis* has suggested that heat stress responsive genes, plant hormones and antioxidant enzymes participate in heat resistance (Kotak et al., 2007).

Heat shock proteins (Hsps) are molecular chaperones that stabilize protein structure and protect the cytoplasmic membrane by mediating the folding, assembly, translocation and degradation of proteins and redundant polypeptides in a normal cellular environment (Zhang et al., 2013). These proteins also maintain cellular metabolic processes and facilitate survival in extreme environments. According to protein molecular weight, the plant Hsps include five subfamilies, including Hsp100, Hsp90, Hsp70, Hsp60, and small Hsps (sHsps) (Hu et al., 2009). The sHsps not only respond to physiological stresses such as heat, but also mediate cellular stress responses via crucial interactions with chaperones (Eylesa and Gierasch, 2010). Based on sequence homology, 10–15 sHsps family members are present in plants, and can be divided into six classes (Kirschner et al., 2000). Class I, II and III are localized in the cytosol or nucleus, and other classes are found in plastids, mitochondria, endoplasmic reticulum or peroxisomes (Sun et al., 2002; Basha et al., 2006). Most sHsps expression are very low or not expressed under normal conditions, but are rapidly induced following exposure to extreme environments. Moreover, sHsps are induced in all organisms in response to environmental stresses and during various developmental processes (Dafny-Yelin et al., 2008).

A recent report provided the first direct genetic evidence that *PpHsp16.4* helps to restore osmotic balance and salt stress tolerance, and therefore functions during stress recovery (Ruibal et al., 2013). *OsHsp18.2*, a class II cytosolic protein, is involved in seed vigor, longevity, and aging (Kaur et al., 2015). Overexpression of AtHsp17.8 in lettuce gives rise to resistance phenotypes in face of dehydration and salt stress through modulating ABA-mediated signaling (Kim et al., 2013). The *PtHsp17.8* protein in *Populus trichocarpa* is involved in heat and salt stress tolerance (Li et al., 2016). Overexpression of the rice *sHsp17.7* confers both heat tolerance and UV-B resistance to rice plants (Murakami et al., 2004). The *sHsp17.4* and *sHsp23.8* proteins from tomato may be involved in protection against chilling (Ré et al., 2017), while *ZmHsp16.9*, a cytosolic class I sHsp from maize, confers heat tolerance in transgenic tobacco (Sun et al., 2012). The *AsHsp17* sHsp in creeping bentgrass modulates photosynthesis and ABA-dependent and independent signaling to attenuate the plant response to abiotic stress (Sun et al., 2016). Furthermore, overexpression of *PfHsp21.4* in *Arabidopsis* enhances heat tolerance (Zhang et al., 2014). Several Hsp20 family members have been reported, including 13 sHsp in *Arabidopsis*, 23 sHsp genes in rice, 51 sHsp candidates in soybean, and 35 putative pepper sHsp genes (Scharf et al., 2001; Waters et al., 2008; Ouyang et al., 2009; Lopes-Caitar et al., 2013; Guo et al., 2015).

*Malus sieversii* (Ledeb) Roem., previously been identified as the progenitor of the cultivated apple (*Malus Domestica* Borkh.), is a tertiary relic species (Yan et al., 2008). *M. sieversii* is mainly located in western Xinjiang in China. Due to limited water resources and a dry climate, this species possesses abundant biological diversity and displays excellent resistance. The Xinyuan population grows in a region with a mild and wet climate, whereas in Daxigou of Huocheng, the *M. sieversii* population must endure high annual average temperatures, but populations flourish despite this challenge. Thus, the species likely harbors valuable genes that are worth mining and analyzing. Although systematic genome sequencing of *M. sieversii* has not yet been performed, a complete genome sequence will provide valuable resources for understanding this species in the future.

In the present work, 12 candidate Hsp20 genes in M. sieversii were identified through bioinformatics analysis and characterized by analysis of sequence features, phylogenetic relationships, and expression patterns. Transcriptome high-throughput sequencing (RNA-Seq) data identified *MsHsp16.9* as a putative heat stress-induced sHsp, and its physiological roles and molecular mechanisms were investigated by overexpression in *Arabidopsis*. The results demonstrated that *MsHsp16.9* encodes a protein chaperone that positively regulates antioxidant enzyme activity and ABA-dependent and independent signaling to attenuate damage following adverse environmental stresses.

## Materials and methods

### Plant materials and RNA sequencing

We collected material from *M. sieversii* from Daxigou of Huocheng (T3) and Xinyuan population (T7) of Xinjiang at the same time in May 2014 (Table S1). Leaves were immediately frozen in liquid nitrogen and stored at −80°C until needed. Based on the optimal cetyltrimethyl ammonium bromide (CTAB) extraction method, total RNA from equal mixed samples (ten individual plants) was extracted using LiCl purification (Meisel et al., 2005). The RNA purity was evaluated with Nanodrop 2,000 Spectrophotometer (Thermo Fisher Scientific, USA). After RNase-free DNase I treatment (New England BioLabs, USA) to remove residual DNA, cDNA library construction and Illumina HiSeq2500 sequencing were performed by Biomarker Technologies Co., Ltd. (Beijing, China).

### Isolation of DEGs and *MsHsp20* genes under heat condition

EBSeq software (an empirical Bayes hierarchical model for inference in RNA-seq experiments) was applied to perform differential expression genes (DEG) analysis. The process utilizes the well-established Benjamini-Hochberg method, which was corrected for significance of *p*-values to generate adjusted *p*-values. During screening, the false discovery rate (FDR) was ≤0.01 and the fold change (FC) was ≥2 (Leng et al., 2013). Combined with the transcriptome sequencing results, *MsHsp20* candidates were identified by alignment with the *M. domestic* genome (https://blast.ncbi.nlm.nih.gov/Blast.cgi).

### Conserved domain analysis and phylogenetic classification of *MsHsp20* genes

The MEME Suite version 4.11.3 (http://meme-suite.org/tools/meme) was used to confirm conserved domains of the *MsHsp20* protein. The full amino acid sequences of Hsp20 members from *M. domestica, Pyrus bretschneideri*, and *Prunus mume* were obtained from NCBI. Gene IDs are shown in Table S3. MEGA 6.0 software was used to construct an unrooted neighbor-joining phylogenetic tree based on 1,000 bootstrap test replicates, pair-wise deletion and a Poisson model.

### Vector construction

Based on RNA-Seq data, the full-length open reading frame of *MsHsp16.9* was PCR-amplified using primers containing the restriction sites *Nco*I and *BstE*II (Table S2). PCR products were firstly cloned into the pEASY-T1 vector and sequenced (TransGen Biotech, China). Following enzyme digestion, the *MsHsp16.9* fragment was then inserted into the binary plant vector pCAMBIA3301 under the control of the CaMV35S constitutive promoter (Figure S1). Recombinant vectors were subsequently transformed into *Agrobacterium tumefaciens* strain LBA4404 using standard heat-shock method.

### Generation of transgenic *Arabidopsis* plants by *Agrobacterium*-mediated transformation and molecular characterization of variants

*Arabidopsis* (Ecotype Columbia) transformation was carried out by the floral dip method (Clough and Bent, 1998). T4 homozygous transgenic progeny lines were obtained through phosphinothricin-resistant screening and molecular identification. The CTAB method was used to extract genomic DNA from young leaves (Ahmed et al., 2009). Positive pCAMBIA3301 transgenic lines were detected based on the sequences of the 35S promoter of pCAMBIA3301 (Table S2). Primer sets used for the p35S::*MsHsp16.9* transgenic lines recognized the forward sequence of the 35S promoter and the reverse sequence of *MsHsp16.9* (Table S2).

### Stress treatment

To analyze heat tolerance in *Arabidopsis* plants, consistent seedlings were chosen for stress treatment. At 5 days, plantlets in solid 1/2 MS medium were subjected to heat shock at 45°C for 3 h. Eight-leaved plantlets and bolting date seedlings were cultured in a growth chamber at 22 ± 2°C with treatment at 45°C for 16 or 48 h (light intensity = 150 μmol m^−2^ s^−1^). All treatments were under a 16:8 h photoperiod. When all plants showed symptoms of severe wilting, plants were moved back to the normal growth environment.

### Determining physiological indices

After heat stress, we estimated the survival rate and root length of 5-day plantlets. The activity of superoxide dismutase (SOD), peroxidase (POD), and catalase (CAT) was determined, as was the malondialdehyde (MDA) content, in eight-leaved plantlets using 752-UV spectrophotometry under heat stress and normal conditions (Wei et al., 2016). After recovering normal growth of florescence, *Arabidopsis* growth indicators including the rosette diameter, stem length, and the size of the silique were measured and analyzed.

### *In Vivo* localization of H_2_O_2_ and O_2_-

Histochemical staining with 3,3-diaminobenzidine (DAB) or nitro-blue tetrazolium (NBT) was performed to analyze the production of H_2_O_2_ and O_2_- (Shi et al., 2010). O_2_- was measured as described previously (Wei et al., 2016), and H_2_O_2_ levels were measured according to the instructions supplied with the H_2_O_2_ Assay Kit (KeyGEN BioTECH, China).

### Quantitative real-time PCR

PCRs contained 10 μl of SYBR I (SYBR Green qPCR kis, Roche) and reactions was performed using iQ5.0 (Bio-Rad, USA) real-time detection system. The reference genes, *MsActin* and *AtActin*, were used as endogenous controls for *M. sieversii* and *Arabidopsis*, respectively. iQ5.0 Optical System Software version 2.1 was used for collecting the data. The 2-ΔΔCt method was used to calculate relative gene expression levels from three biological replicates.

### Statistical analysis

All data are presented as the means ± standard error (SE) of at least three replicates. The Student's *t*-test was used to test the significance of differences between the control plants and transgenic lines. Asterisks (^*^, ^**^, or ^***^) indicate a significant difference between the controls and transgenic plants at *P* < 0.05, 0.01, or 0.001, respectively.

## Results

### Transcriptome sequencing of *M. sieversii* under heat stress

**Figure 1:**
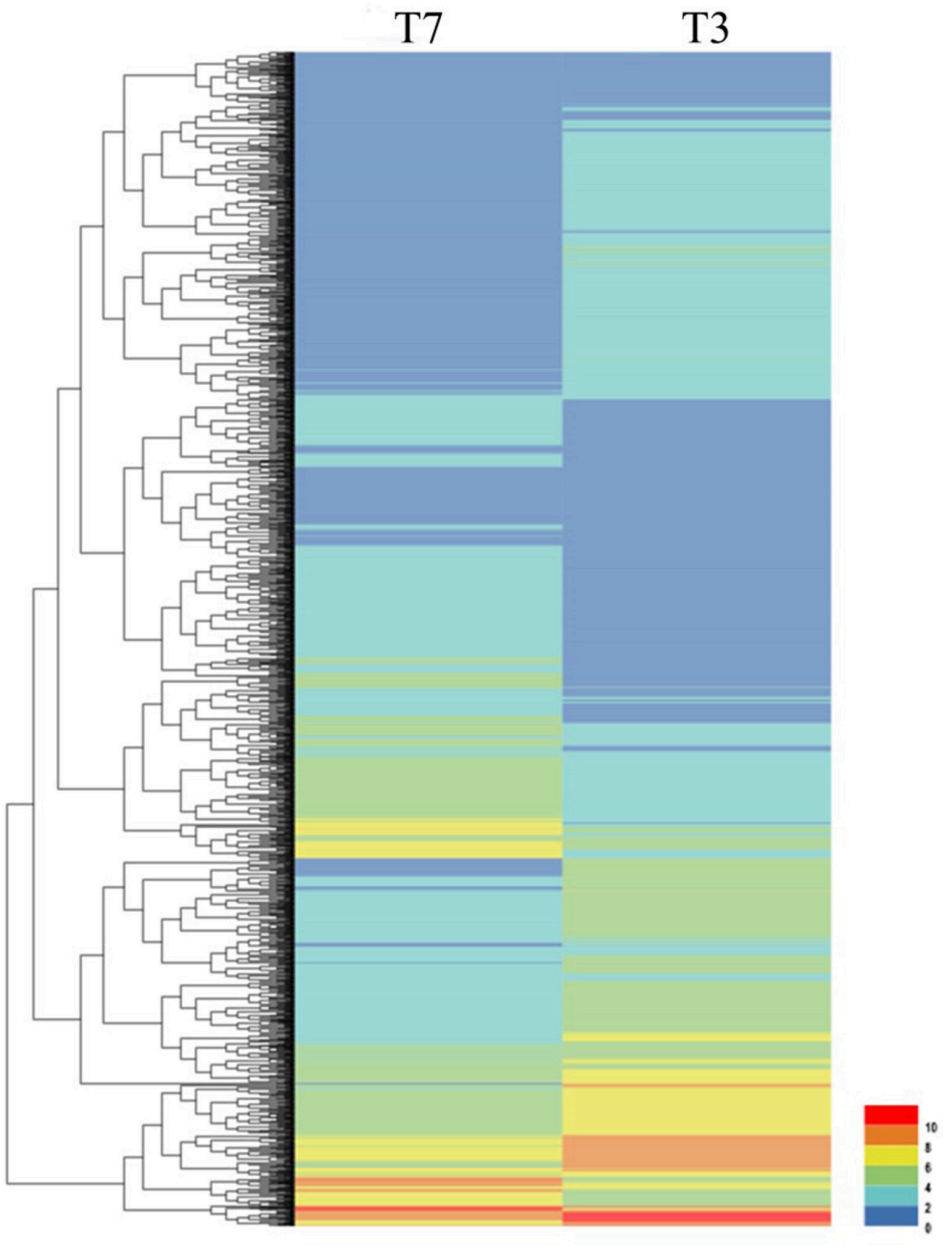
Clustering analysis of DEGs in T7 and T3 *Malus sieversii* based on their expression profiles obtained by RNA-Seq. T7, Xinyuan *Malus sieversii*; T3, Daxigou *Malus sieversii*. The color scale corresponds to the log2 (FPKM) values of genes in various samples.

**Figure 2:**
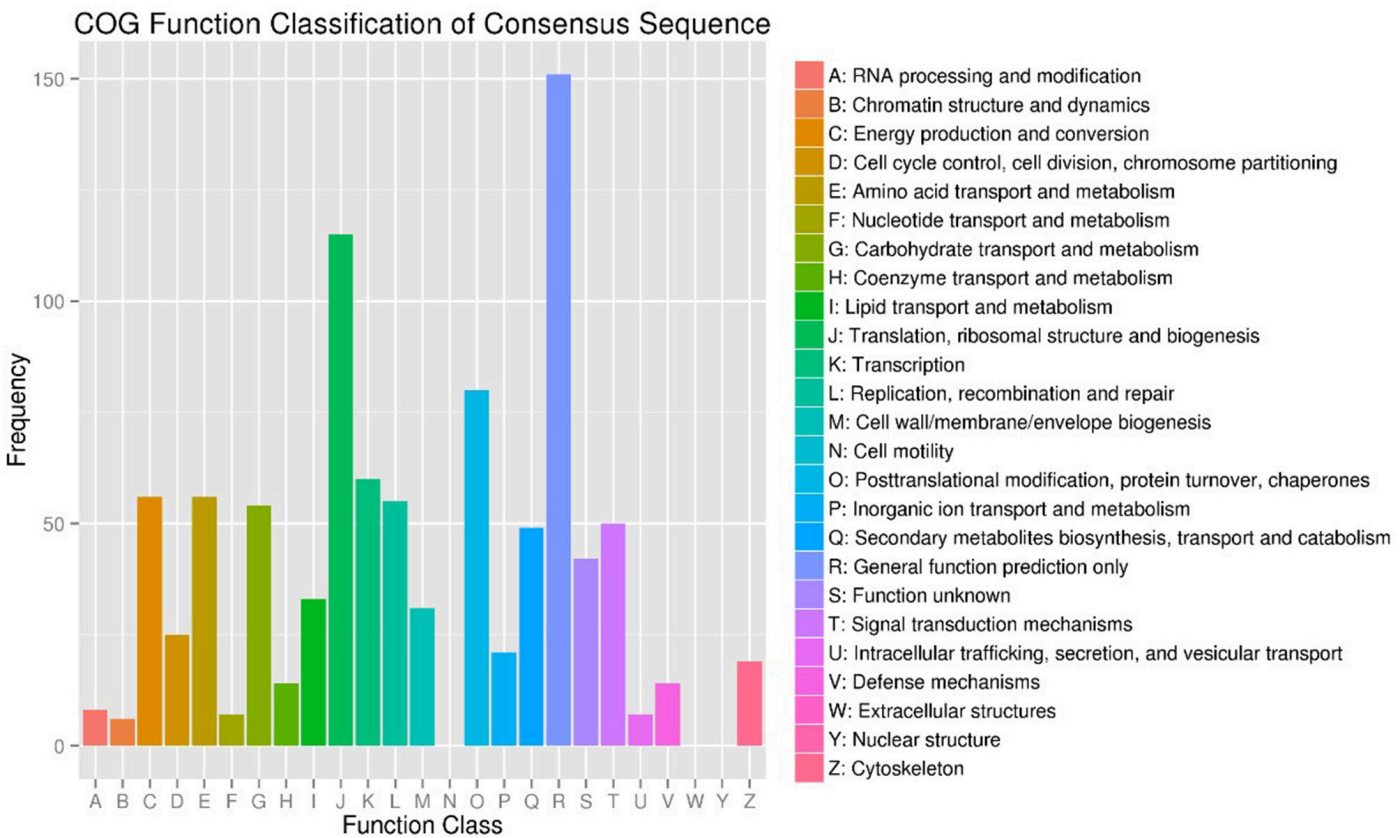
COG Function Classification of DEGs in T7 and T3 *Malus sieversii* based on their expression profiles obtained by RNA-Seq. T7, Xinyuan *Malus sieversii*; T3, Daxigou *Malus sieversii*.

**Figure 3:**
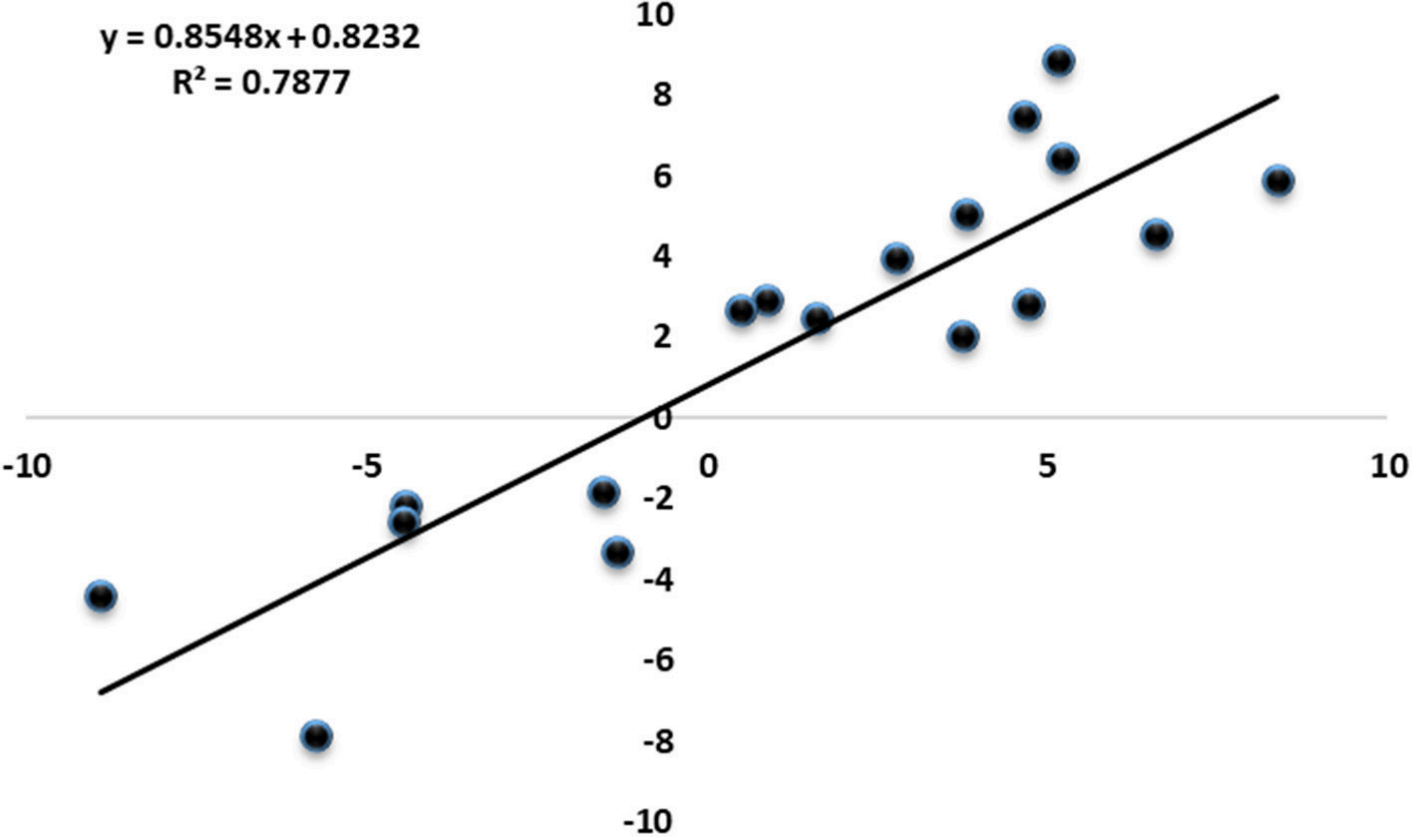
Correlation of expression fold changes determined by RNA-Seq (x-axis) and qRT-PCR data (y-axis).

To investigate the genes response to heat stress, we compared the expression pattern of T7 and T3, and the transcriptome data has deposited in the SRA database of NCBI (T3 accession number: SRR6027256; T7 accession number: SRR6027926). Mixed RNA samples were used to generate a cDNA library, yielding 25,641,037 and 28,821,560 clean reads for T7 and T3, respectively. The guanine–cytosine contents (GC) was 47.64 and 47.55%, and two Q30 base percentage was not less than 85.01% (Table S4). 2,518,193 contigs, 26,013 transcripts and 62912 unigenes were identified on the basis of Trinity assembly (Table S5). We analyzed the expression of the unigenes in T7 and T3. A total of 2728 DEGs were identified via clustering analysis (Figure 1) and divided into 25 groups according to the COG classification (Figure 2). In all COG categories, posttranslational modification, protein turnover and chaperones were located in the third place. In order to confirm the validity of the RNA-Seq data, we selected 18 genes at random that were significantly upregulated and downregulated in T7 and T3 for further analysis by quantitative RT-qPCR (Table S2). The relative expression levels of these genes were similar to those determined from the respective RNA-Seq data. A high correlation (*R*^2^ > 0.7877) was found between the RT-qPCR and RNA-Seq results (Figure 3, Supplementary Data Sheet 1), confirming the accuracy of the RNA-Seq data.

### Screening and characterization of putative *MsHsp20* family members

A total of 12 candidate *Hsp20* gene sequences were identified from RNA-Seq DEG data, which were annotated using Swiss-Prot (Apweiler et al., 2004), COG (Tatusov et al., 2000), KOG (Koonin et al., 2004), KEGG (Kanehisa et al., 2004), Pfam (Finn et al., 2014) GO (Ashburner et al., 2000), and non-redundant (Nr) annotation databases with BLAST parameters *E* ≤ 10^−5^ and HMMER parameters *E* ≤ 10^−10^ (Table S3). The identified genes showed significant differential expression between T7 and T3, and up-regulation in T3 indicated a common response. Among these genes, the log2FC value was greater than 2 for all except c50641.graph_c0, c55233.graph_c0, and c56990.graph_c0 (Table S3). The C61701.graph_c0 gene, encoding a 16.9 kDa class I Hsp, was expressed highly at all times in both T3 andT7 (Table S3).

**Figure 4:**
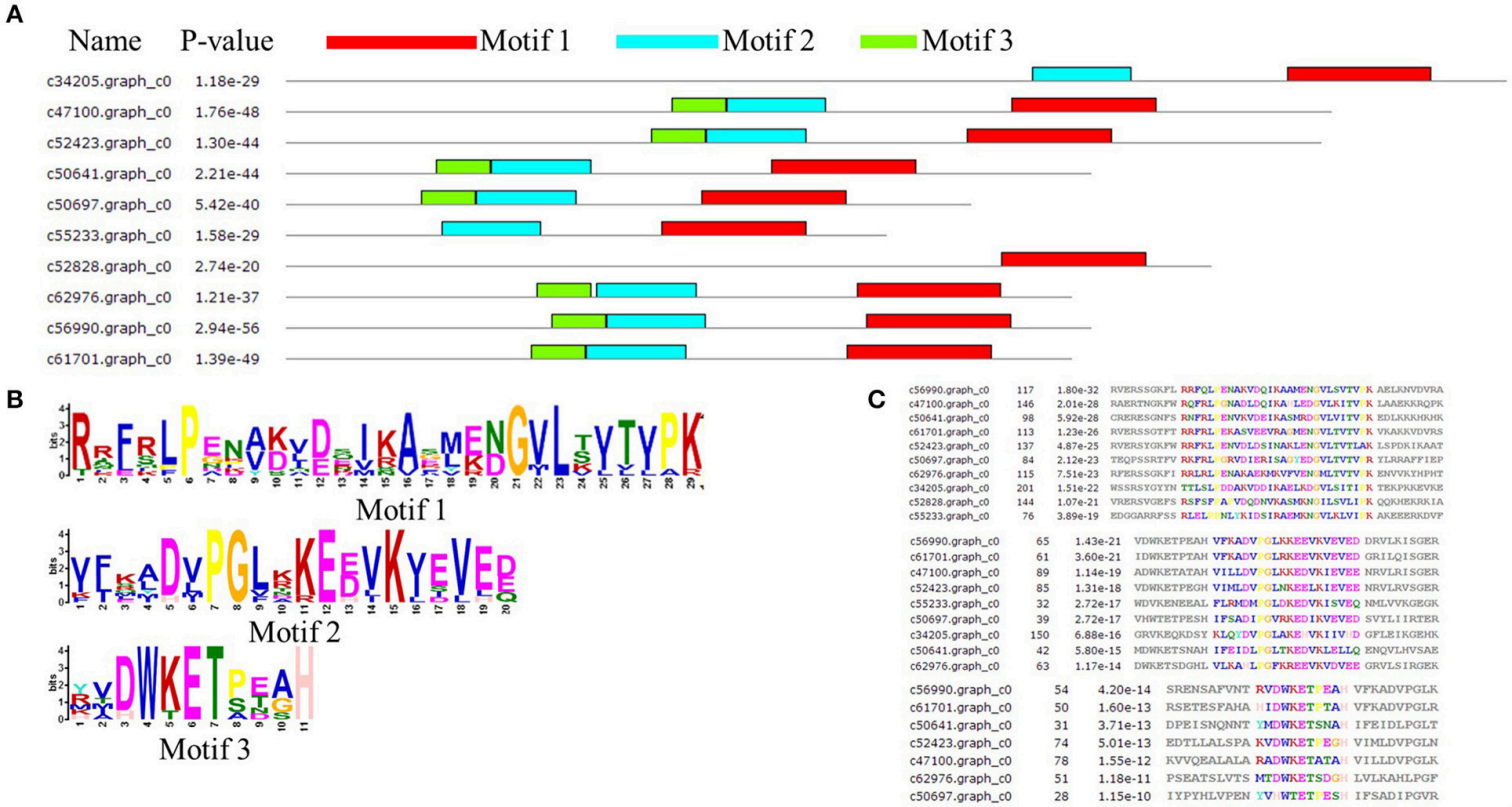
Architecture of predicted *Hsp20* sequences in *Malus sieversii*. **(A)** Structural analysis of the *MsHsp20* protein. The conserved α-crystallin domain (ACD) of *sHsp20* is shown in pink. Distribution of conserved motifs in *MsHsp20* proteins were identified using MEME software. Putative motifs are represented by different colors. The names of all members and combined *p*-values are shown on the left side of the figure. **(B)** Hidden Markov model logos obtained using MEME. **(C)** Conserved motif sequences of *Hsp20* genes in *Malus Sieversii*.

The amino acid sequence of *MsHsp20* proteins ranged from 136 (>c50697.graph_c0) to 243 (>c34205.graph_c0) residues in length, and all possessed a conserved alpha-crystallin domain (ACD) except for >c30087.graph_c0 and >c33770.graph_c0 based on analysis by MEME (Figure 4A). In addition to >c34205.graph_c0, >c55233.graph_c0 and >c52828.graph_c0, all *MsHsp20* members share similar motif, motif1, motif 2 and motif 3 regions, and both nucleotide and amino acid sequences are highly conserved (Figures 4B,C). These motifs are conserved in *M. sieversii*, and although the functions of these motifs are not yet clear, the presence of similar conserved motifs likely reflects common functions.

### Phylogenetic analysis of the *MsHsp20* family

**Figure 5:**
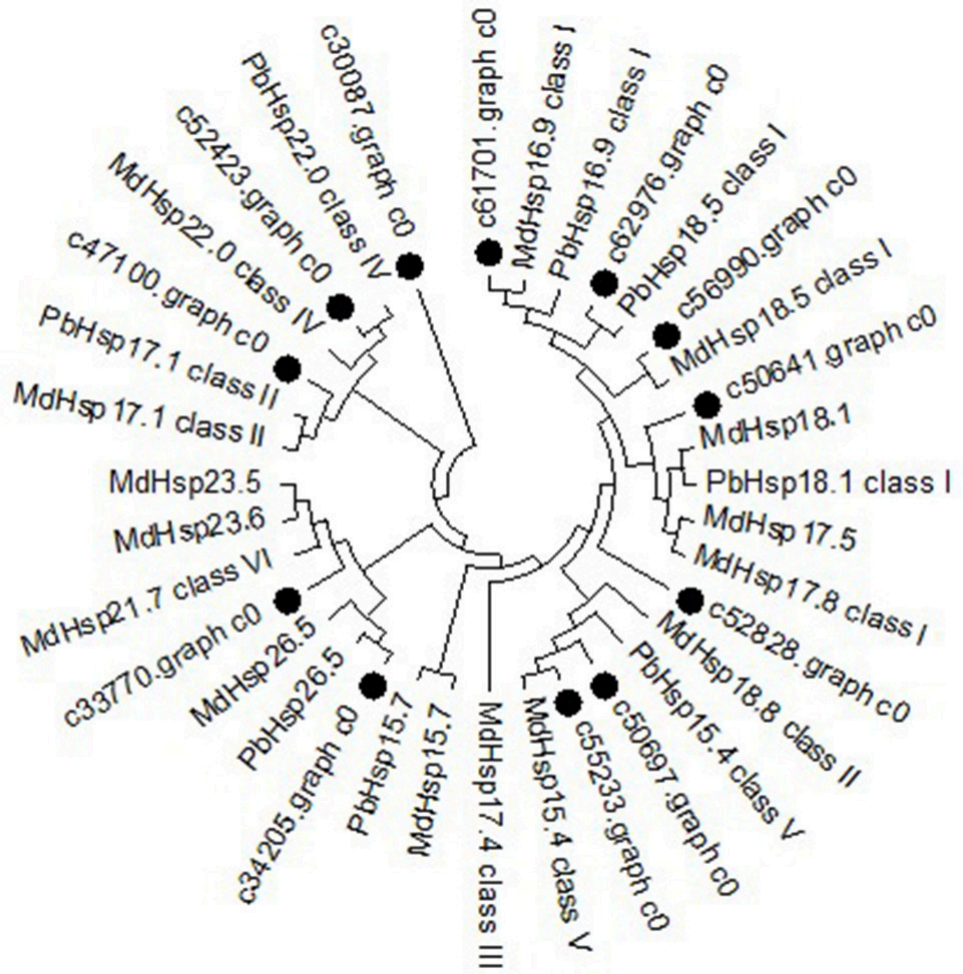
Phylogenetic tree of *Malus sieversii* Hsp20 proteins. The tree was constructed by aligning the complete protein sequences of Hsp20s from the following species: *Malus sieversii* (•), *Malus x domestic* (Md), *Pyrus x bretschneider* (Pb), *Prunus mume* (Pm).

To further determine the classification characteristics among *MsHsp20* proteins, a phylogenetic tree was constructed with well-supported bootstrap values (1000 replicates), which contained *MdHsp20* and *PbHsp20* full-length protein sequences. This resulted in the identification of six distinct clusters (classes I–VI; Figure 5). Hsp20 proteins from *M. domestica, P. bretschneideri*, and *P. mume* are present in all classes, and *M. sieversii* sequences are found in all classes. c61701.graph_c0 has high conservation and belongs to class I, together with c56990.graph_c0, c62976.graph_c0, c47100.graph_c0, and c50641.graph_c0. *MsHsp20* proteins in classes I and III include c52828.graph_c0, c33370.graph_c0, and c50697.graph_c0, while c55233.graph_c0 and c52423.graph_c0 represent class IV and V, respectively. Other members belong to Class VI.

### Expression of *MsHsp20* family members in response to heat stress

**Figure 6:**
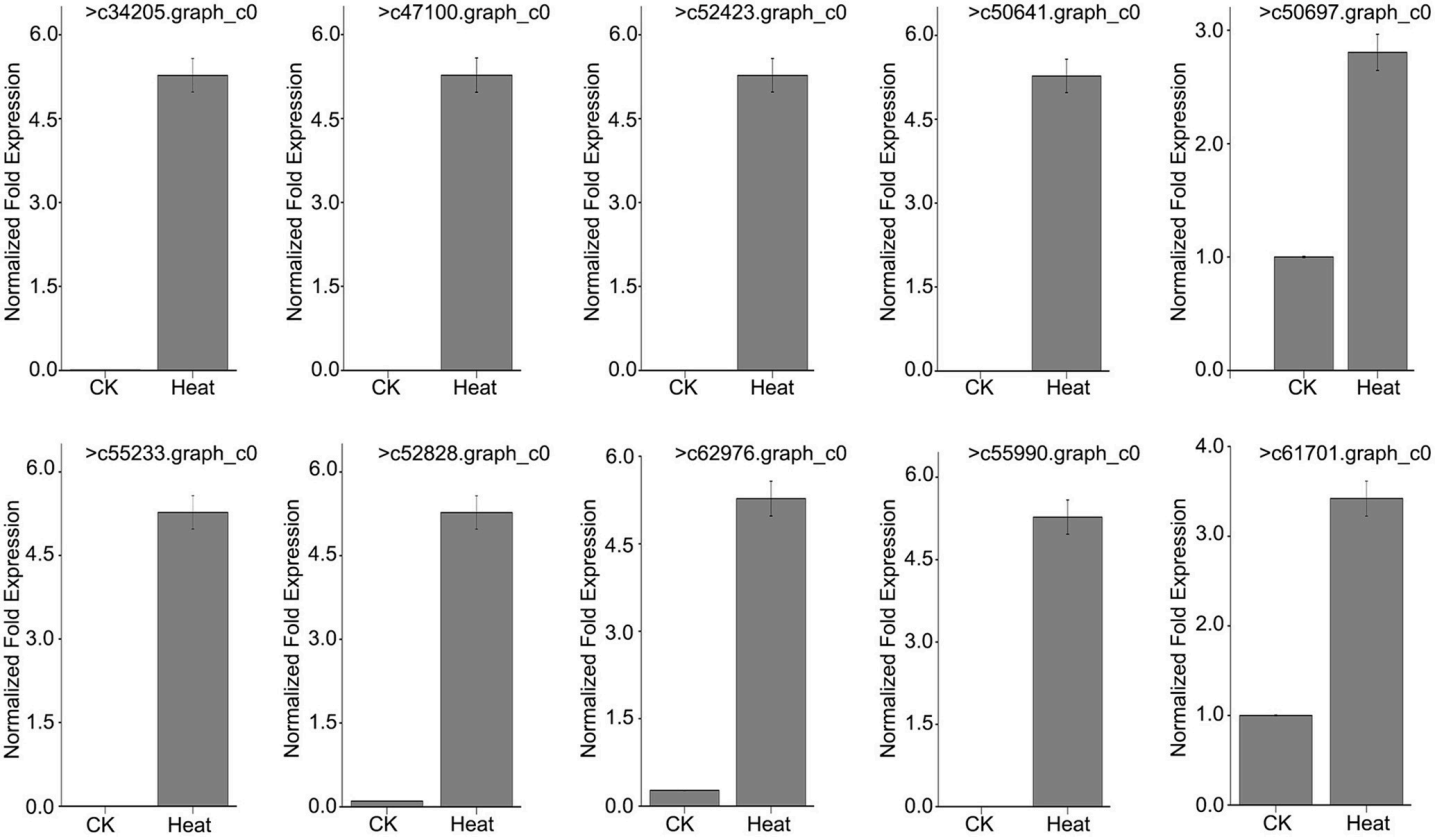
Expression pattern of 10 *Malus sieversii Hsp20* genes under control check (CK, 25°C) and heat treatment (42°C) conditions.

To further verify that *MsHsp20* members are involved in plant responses to environmental stresses, we used RT-qPCR to determine the expression profile of 10 *MsHsp20* genes in aseptic seedlings (grown at 25 and 42°C) in response to heat shock (Table S6; Figure 6). The expression levels of the 10 *MsHsp20* genes appeared rather low at 25°C, but all were significantly up-regulated in response to heat stress at 42°C (Figure 6). This result indicated that these *MsHsp20* genes could respond to heat stress. The primers used are listed in Table S2. To visualize the gene expression patterns, we created a heat map in which each line represents genes with significant differential expression. (Figure S2) Compared with other genes, the expression of >61,701.graph_c0 and >50,697.graph_c0 was relatively lower (Figure S2).

### Generation and molecular analysis of *MsHsp16.9* transgenic plants

**Figure 7:**
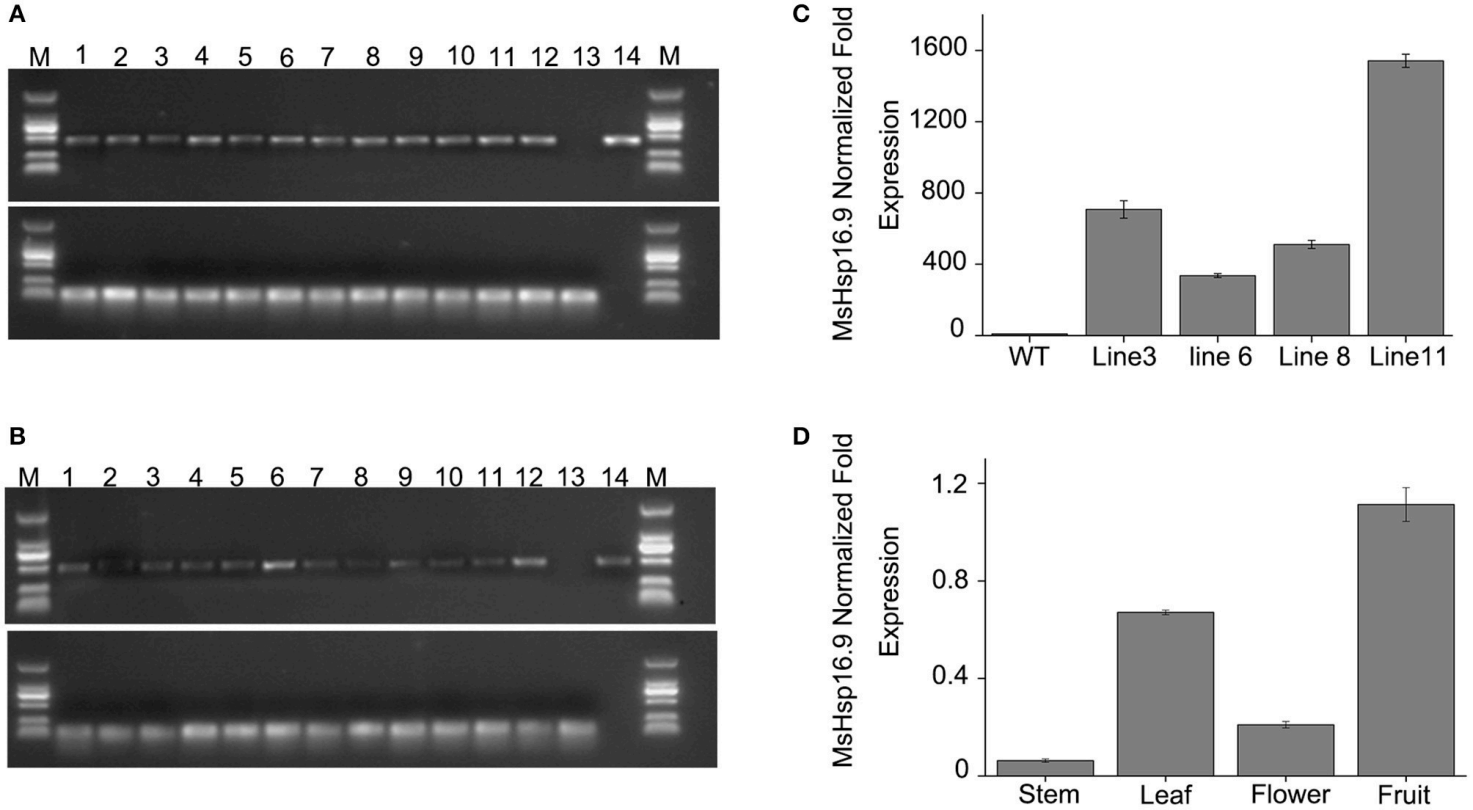
Screening and identification of pCAMBIA3301 and *MsHsp16.9* overexpression positive plants. **(A)** PCR analysis of primary transformants using specific primers for the pCAMBIA3301 vector. Lanes 1 and 16, size makers; lanes 2–13, DNA from putitive transformants; lane 14, untransformed control; lane 15, pCAMBIA3301 positive control. The actin gene was used as a control. **(B)** PCR analysis of primary transformants using specific primers for 35S::*MsHSP16.9*. Lanes 1 and 16, size markers; lanes 2–13, DNA from putitive transformants; lane 14, untransformed control; lane 15, P35s::*MsHSP16.9* positive control. The actin gene was used as a control. **(C)** qRT-PCR analysis of T2 transformants using quantified primers for 35S::*MsHSP16.9*. WT, untransformed control; Lines 3, 6, 8, and 11, T2 transformant positive lines. **(D)** *MsHsp16.9* tissue-specific expression in *Arabidopsis*.

To examine the role of *MsHsp16.9* in *Arabidopsis*, we constructed transgenic plants harboring p35S::*MsHsp16.9* (Figure S1). We selected pCAMBIA3301 vector control transgenic lines by spraying Basta and molecular identification (Figure 7A). Similarly, 12 positive *MsHsp16.9* overexpression lines were initially screened with Basta, and PCR was then performed with specific primers, resulting in the amplification of a 471 bp *MsHsp16.9* fragment (Figure 7B). We performed RT-qPCR analysis using cDNA from p35S::*MsHsp16.9* lines 3, 6, 8, and 11. Compared with untransformed control plants (WT), expression of *MsHsp16.9* was 400–1500-fold higher. We chose relatively high expressing lines (3, 8, and 11) for subsequent experiments (Figure 7C). Tissue localization of *MsHsp16.9* expression was then investigated, and expression was particularly high in the leaves and pods, and lower in stems and flowers (Figure 7D).

### Overexpression of *MsHsp16.9* weakens plant sensitivity to heat stress associated with enhanced survival rate in 5-day plantlets

**Figure 8:**
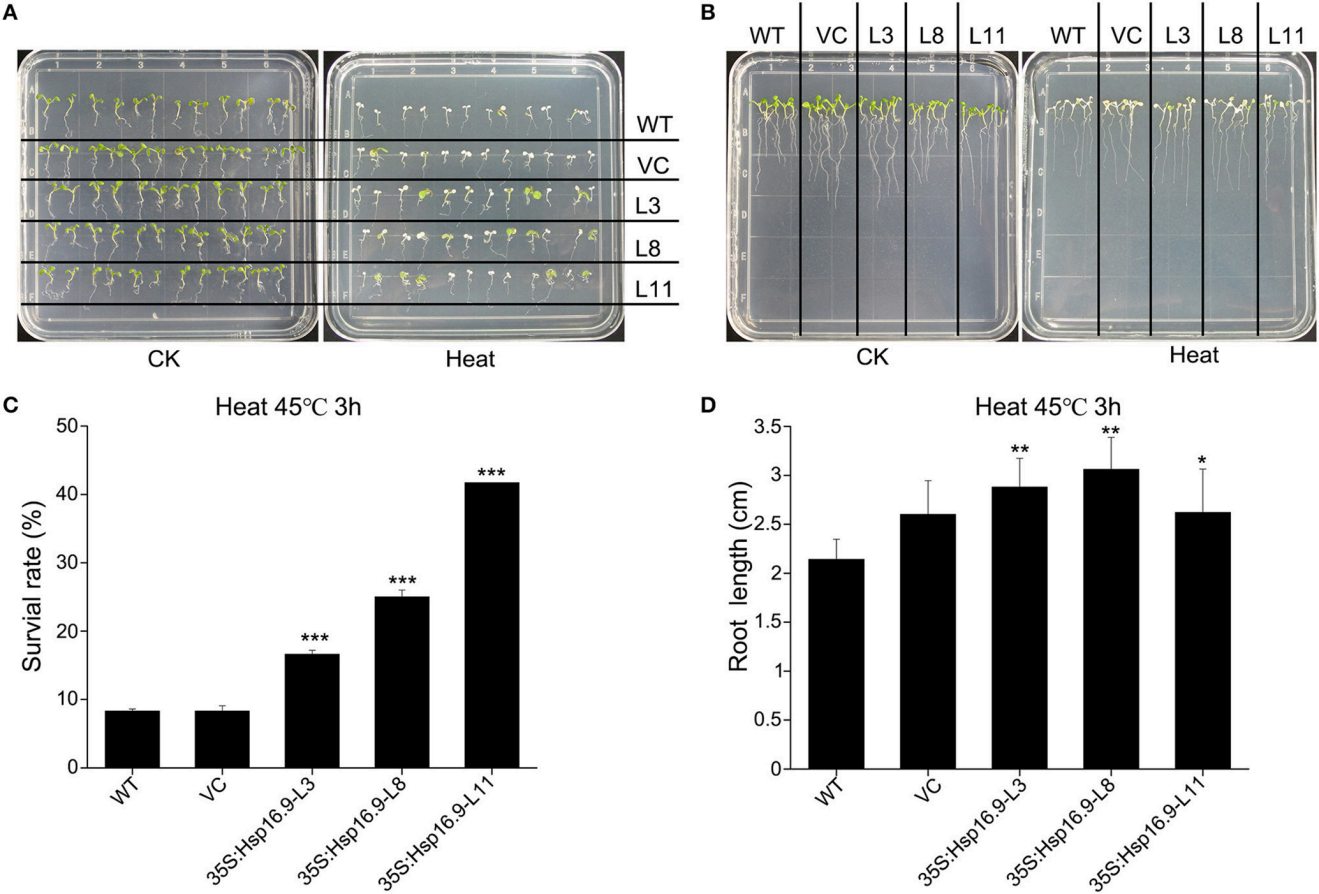
Growth of *MsHsp16.9*-overexpressing plants following heat stress at 45°C for 3 h. **(A)** Growth status of wild-type (WT) and transgenic seedlings under control check (CK) and heat stress conditions. **(B)** Root growth status of WT and transgenic seedlings under control check (CK) and heat stress conditions. **(C)** Survival rate of WT and transgenic seedlings following heat stress. **(D)** Root length of WT and transgenic seedlings following heat stress. Bars represent the mean ± SE of three independent experiments. ^*^, ^**^, ^***^Represent significant differences at *p* < ^#^0.05 and *p* < ^#^0.01 and *p* < ^#^0.001 compared with WT plants.

**Figure 9:**
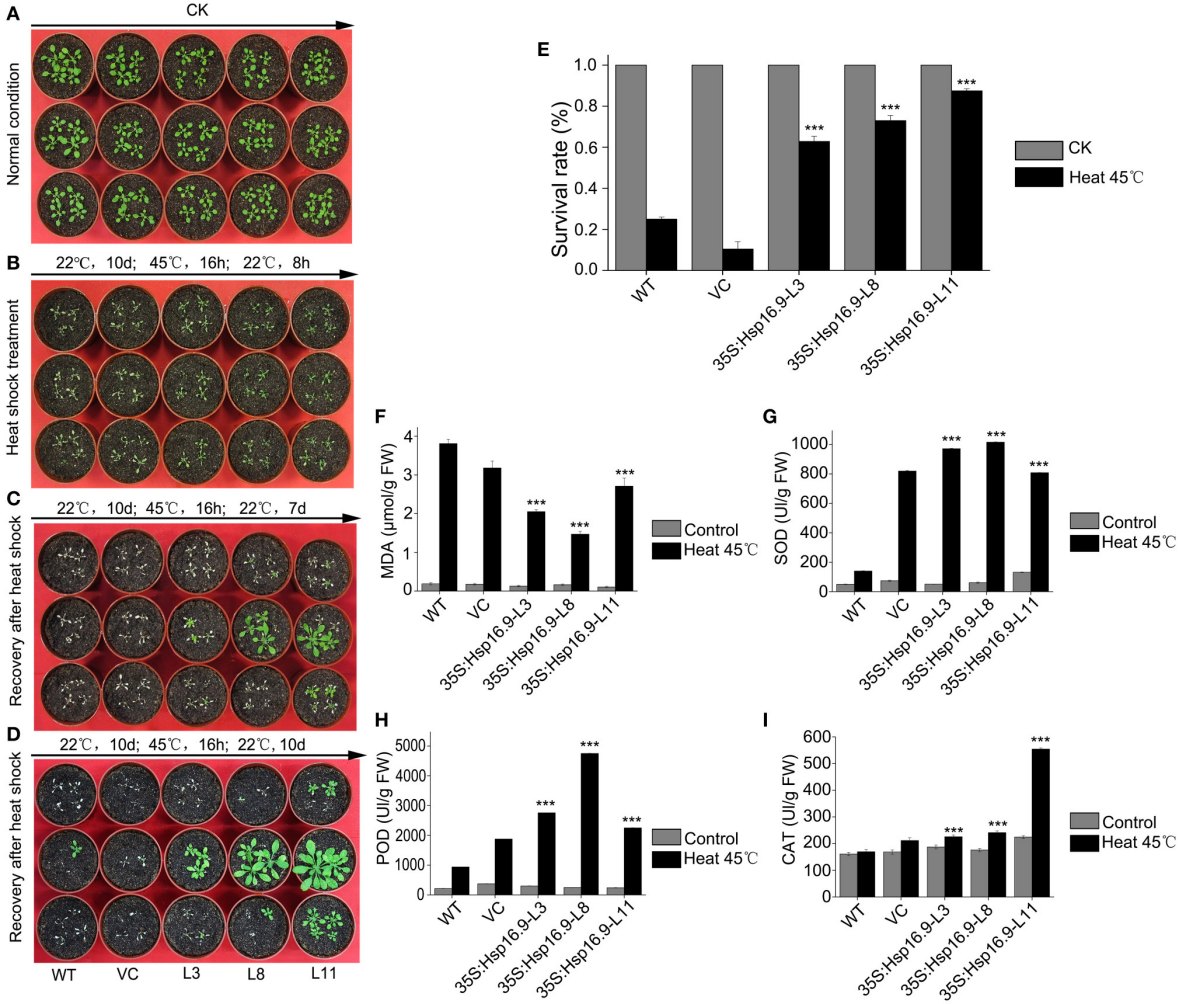
Growth and biochemical analysis of *MsHsp16.9*-overexpressing plants after heat stress at 45°C for 16. **(A)** Growth status of control and transgenic *Arabidopsis* plants under normal conditions. **(B)** Growth status of control and transgenic *Arabidopsis* plants after an 8 h recovery following 45°C heat stress. **(C)** Growth status of control and transgenic *Arabidopsis* plants after a 7-day recovery following 45°C heat stress. **(D)** Growth status of control and transgenic *Arabidopsis* plants after a 10-day recovery following 45°C heat stress. **(E)** Survival rate of control and transgenic *Arabidopsis* plants under normal conditions and after 45°C heat stress for 16 h. **(F–I)** MDA, SOD, POD and CAT levels in control and transgenic *Arabidopsis* plants under normal conditions and after 45°C heat stress. Bars represent the mean ± SE of three independent experiments. ^***^Represent significant differences at *p* < ^#^0.001 compared with WT plants.

We observed the growth of *MsHsp16.9* transgenic *Arabidopsis* plants and compared them with WT plants and pCAMBIA3301 vector control plants in 1/2 MS medium. Three overexpression lines showed no obvious changes compared with the control (Figures 8A, 9B). To investigate whether *MsHsp16.9* expression was associated with heat tolerance, we subjected transgenic plantlets together with WT and pCAMBIA3301 transgenic plants possessing 2–4 leaves to heat at 42°C for 3 h (Figures 8A, 9B). The survival rate of the three lines of transgenic plants was 16, 25, and 41%. However, survival of WT and pCAMBIA3301 transgenic plants was only 8% (Figure 8C). In addition, the average root length of transgenic plants was longer than control plants (Figure 8D). These phenotypic differences suggest overexpression of *MsHsp16.9* may weaken the plant sensitivity to heat stress.

### *MsHsp16.9* overexpression enhances survival and increases protective enzyme activity under heat stress

To investigate whether heterogenous expression of the *MsHSP16.9* gene alters transgenic plants heat responses, transgenic and control *Arabidopsis* with 8 to 10 leaves were incubated at 45°C for 16 h (Figure 9A). The results showed that transgenic lines were less sensitivity than WT and VC control plants, and displayed less withering, indicating more stable turgor pressure and slower onset of senescence (Figure 9B). After 7 days recovery from the heat treatment, transgenic plants exhibit varying degrees of growth and some leaf death. By contrast, almost all WT and VC plants that developed leaves had died (Figure 9C). At 10 days after heat treatment, surviving WT and VC plants had begun to grow again, whereas most transgenic lines had completely restored growth by this stage and were close to the bolting stage (Figure 9D). The plant survival rate was further analyzed, and the results showed that both control and transgenic plants under normal growth conditions had a similar growth rate, but control plants was less than that of transgenic plants under heat stress (Figure 9E). Additionally, we investigated oxidase system activity and membrane damage. The activity of SOD, POD and CAT was clearly increased in transgenic plants (Figures 9G–I). Consistent with this, elevated MDA levels were found in control plants under stress conditions, compared with the lower levels observed in transgenic lines (Figure 9F).

### Overexpression of *MsHsp16.9* alleviates heat-induced ROS damage and maintains growth

**Figure 10:**
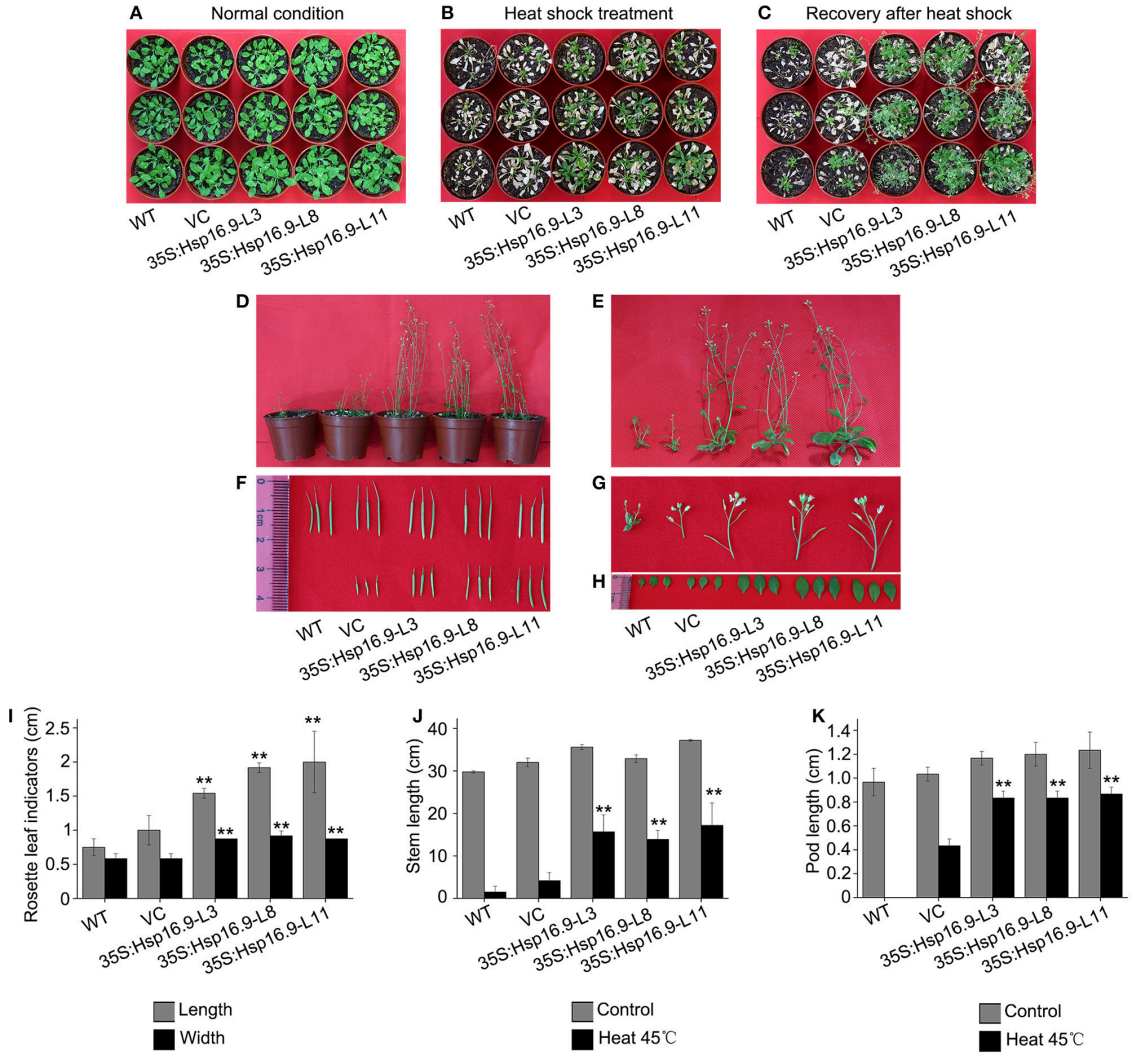
Growth status of WT, VC and *MsHsp16.9*-overexpressing *Arabidopsis* plants following 45°C heat stress for 48 h during the reproductive stage. **(A)** Growth status of control and transgenic plants under normal conditions. **(B)** Growth status of control and transgenic plants after 45°C heat stress for 48 h. **(C)** Growth status recovery of control and transgenic plants after 45°C heat stress. **(D)** Recovery of whole control and transgenic plants. **(E–H)** Recovery of the stem, silique, flower, and rosette leaf tissue of control and transgenic plants. Survival rate of WT and transgenic plants under normal conditions and after 45°C heat stress for 16 h. **(I–K)** Length of rosette leaf, stem, and silique indicators in control and transgenic plants. Bars represent the mean ± SE of three independent experiments. ^**^Represent significant differences at *p* < 0.01 compared with WT plants.

To further assess the effects of heat treatment on reproductive growth, WT, VC, and *MsHsp16.9*-overexpressing transgenic bolting stage plants were incubated at 45°C for 48 h then recovered until the full blossom stage (Figures 10A–C). Rosette leaf diameter, stem length, and pod length were measured in WT, VC and transgenic seedlings after recovery. Regarding the overall plant structure, control and transgenic plants showed different degrees of bleaching and flowering time delay. Furthermore, transgenic plants had formed pods, while control plants had lost nearly all reproductive ability (Figures 10F,K). The main reason is that the stem and branches of control plants adopted a dwarf phenotype, leading to the failure of flowering and pollination (Figures 10D,E,G,J). Consistently, the smaller rosette leaves and single blades of WT and VC plants also had an adverse effect on blossoming (Figures 10H,I).

**Figure 11:**
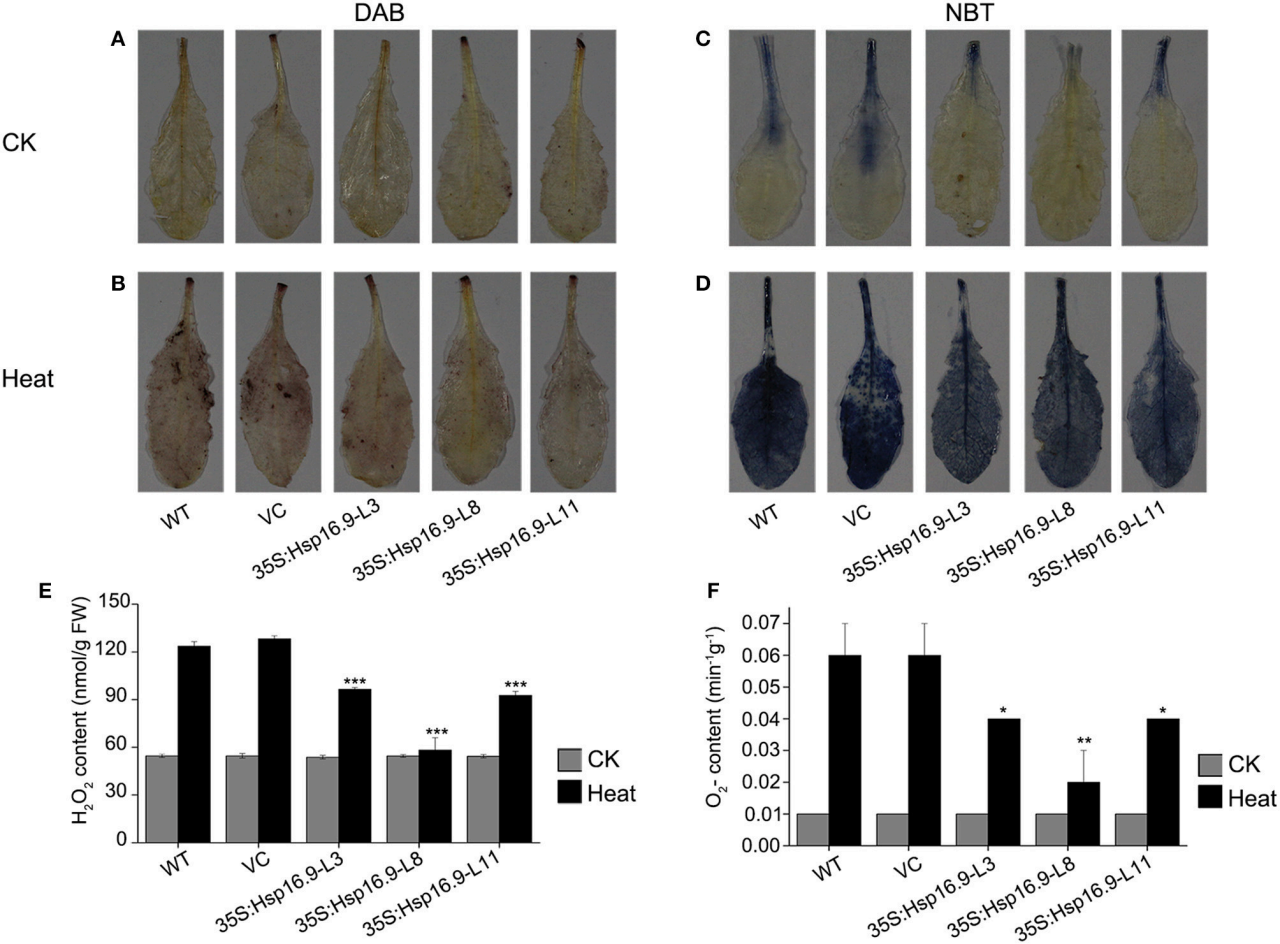
Changes in O_2_- and H_2_O_2_ levels in WT, VC and *MsHsp16.9*-overexpressing *Arabidopsis* plants after 45°C heat stress for 48 h. Heat-stressed leaves were incubated in nitroblue tetrazolium (NBT) or diaminobenzidine (DAB) solution. **(A–C)** Brown staining indicating H_2_O_2_ accumulation under normal and heat stress conditions. **(D–F)** Blue staining indicating O_2_- accumulation under normal and heat stress conditions. Bars represent the mean ± SE of three independent experiments. ^*^and ^**^Represent significant differences at *p* < 0.05 and *p* < 0.01 compared with WT plants. Bars represent the mean ± SE of three independent experiments. ^***^Represent significant differences at *p* < 0.001 compared with WT plants.

Heat stress can increase the generation of reactive oxygen species (ROS), thus we investigated whether transgenic plants accumulated less ROS under heat stress treatment by examining O_2_- and H_2_O_2_ accumulation using NBT and DAB staining, respectively. Under normal growth conditions, DAB and NBT staining was minimal in transgenic and non-transgenic plants (Figures 11A,C). However, staining was increased following heat treatment in all plants, but injury in control plants was clearly more serious than in transgenic plants (Figures 11B,D). Regarding O_2_- and H_2_O_2_, levels of both were higher in WT and VC plants (Figures 11E,F). These results further confirmed the protective role of *MsHSP16.9* in heat stress tolerance.

### *MsHsp16.9* affects ABA synthesis and catabolism and is involved in ABA-mediated signaling

**Figure 12:**
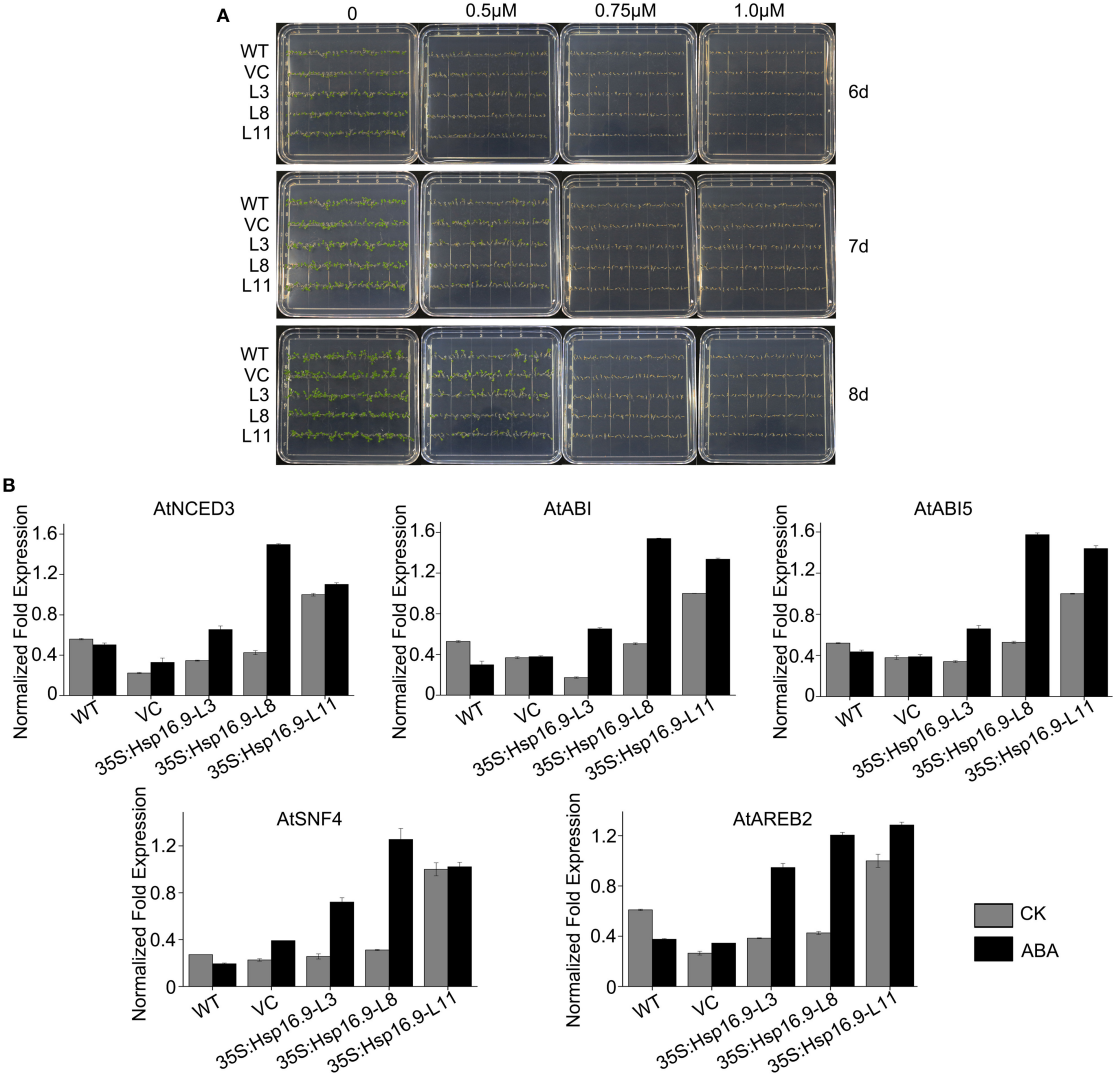
Germination and greening rates of WT, VC and transgenic plants in response to abscisic acid (ABA). **(A)** Seed germination of WT, VC, and *MsHSP16.9* transgenic (TG) plants subjected to 0, 0.5, 0.75, and 1.0 μM ABA at 6, 7, and 10 days after treatment. **(B)** Expression profiles of genes involved in ABA biosynthesis and catabolism in WT and *MsHSP16.9* transgenic plants.

The plant hormone abscisic acid (ABA) is a major regulator of plant growth and a variety of stress environments (Finkelstein et al., 2002; Xiong et al., 2002; Himmelbach et al., 2003). To assess whether the role of *MsHsp16.9* in abiotic stress responses involves ABA, we compared sensitivity to exogenous ABA during germinative and post-germinative growth in WT and transgenic plants. The results showed that *MsHsp16.9*-overexpressing plants did not exhibit an obvious difference in their response to exogenous ABA during germination or growth (Figure 12A). However, the expression of genes in ABA-mediated signaling pathways was distinctly changed compared with WT plants. The 9-cis epoxycarotenoid dioxygenase (NCED) encoded by the AtNCED3 gene can catalyze the limiting step of ABA biosynthesis (Roychoudhury et al., 2013). AtNCED3, together with ABA-responsive gene, AtABI, AtABI5, AtSNF4, and AtABER2, was up-regulated in all transgenic lines compared with the controls under normal and ABA treat conditions (Figure 12B), especially in 35S:*MsHsp16.9*-L11 (TP11). This difference in ABA biosynthetic pathway gene expression between *MsHsp16.9* transgenic and control plants suggests that heterogenous expression of *MsHsp16.9* may increase ABA biosynthesis and accumulation in plants, and raises the possibility that *MsHsp16.9* acts as a positive regulator in ABA signaling in *Arabidopsis*.

### Expression of heat stress-related genes in *MsHsp16.9*-overexpressing transgenic plants

**Figure 13:**
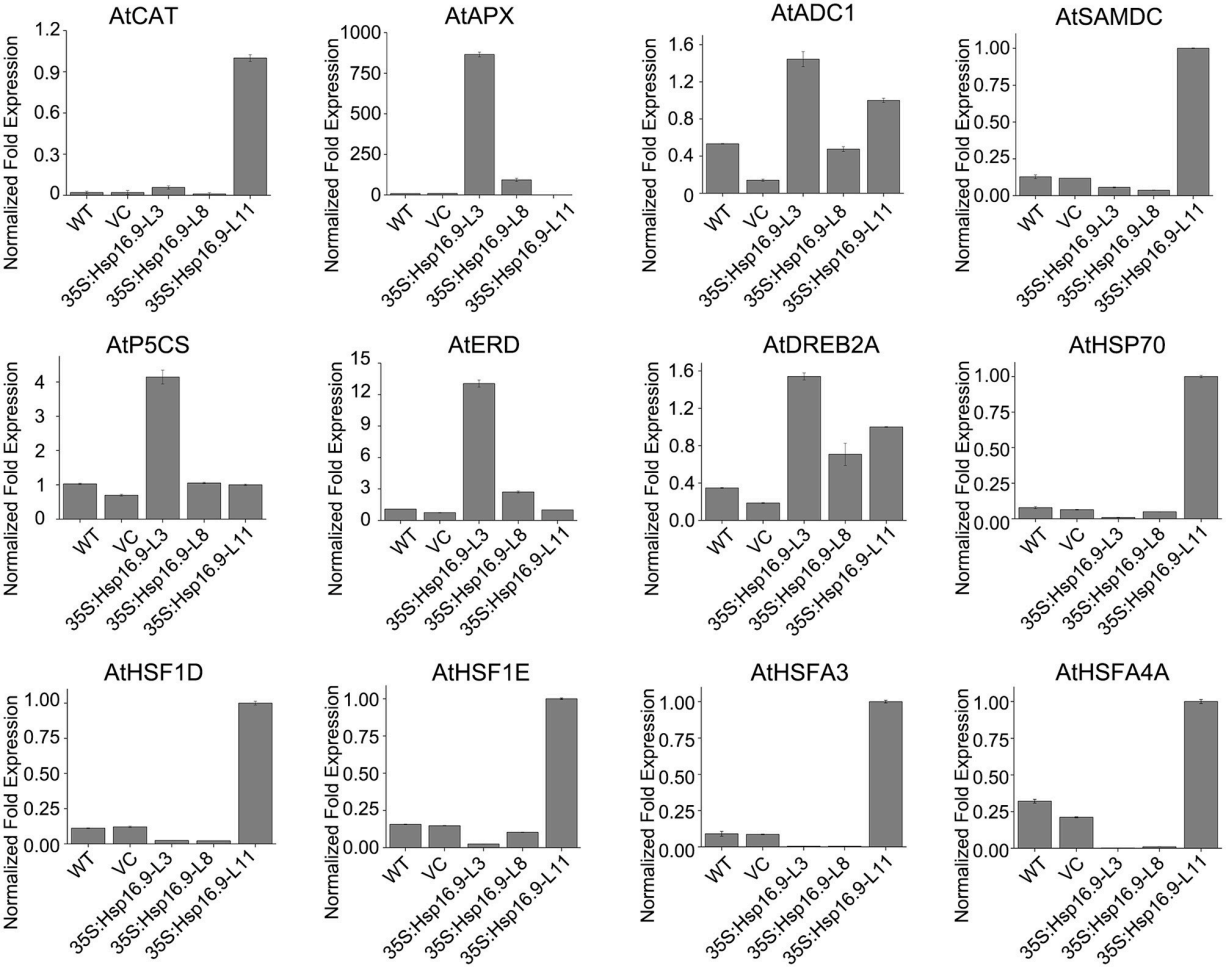
Expression profiles of genes related to *MsHsp16.9*.

To explain the phenotypic characteristic of the *MsHsp16.9* transgenic plants under heat stress and have an insight into the molecular mechanisms of *MsHsp16.9*-mediated regulation of the heat stress response, we focused on heat stress-related genes that are differentially expressed between WT and *MsHsp16.9* transgenic plants. These genes were divided into two groups: regulatory factors and functional proteins. The regulatory proteins mainly included heat-shock factor (HSF) family members (HSF1D, HSF1E, HSFA3, and HSFA4A) as well as the dehydration-responsive element binding protein (DREB2A). All HSF genes were up-regulated in TP11 but not in other lines. The expression of Hsp70 was also consistent with HSF. These results, together with the observed phenotypes and high expression of *MsHsp16.9* in TP11, suggests that *MsHsp16.9* may activate Hsp70 and HSF to maintain protein stability, directly or indirectly protecting cells from damage during high expression. Besides, DREB2A has pivotal effect on regulating the heat response in all transgenic lines including 35S:*MsHsp16.9*-L3 (TP3), 35S:*MsHsp16.9*-L8 (TP8) and TP11 (Figure 13). Regarding functional proteins, the high expression of CAT and ascorbate peroxidase (APX) genes in transgenic lines TP3 and TP11 may explain the observed alleviation of oxidative damage (Figure 13). In addition, arginine decarboxylase (ADC1), S-adenosylmethiomine decarboxylase (SAMDC) and 1-pyrroline-5-carboxylate synthase (P5CS), all associated with secondary metabolism, were up-regulated in transgenic lines under heat stress compared with WT and VC plants (Figure 13). Similar results were observed for the early response to delydration Stress (ERD) gene. These osmotic regulation-associated proteins was crucial for cellular homeostasis and be affected by *MsHsp16.9* under heat stress.

## Discussion

Xinjiang is located in the hinterland of Eurasia and has a complicated geographical structure with tall mountains and basins. The Tienshan mountains act as a boundary, and Xinjiang is divided into southern and northern parts. Due to the influence of westerlies all year round that are associated with the extreme topographical conditions, the north of Xinjiang is more humid than the south. Thus, wild fruit trees are mainly concentrated in northern Xinjiang. *M. sieversii* is the main species complex, and it has evolved strong resistance characteristics. Daxigou in Huocheng (T3) is the site of an 850 hm^2^ wild fruit forest growing at an altitude of 1,180–1,700 m, whereas Xinyuan (T7) has a wild fruit forest covering 700 hm^2^ at an altitude of 1,240–1,650 m. The most significant environmental differences between the two areas is the annual average temperature, which is 9.0°C in T3 and 8.1°C in T7. Although there is strong evaporation and low precipitation in T3, *M. sieversii* still manages to grow a prosperous root system, and achieve vigorous growth and strong resilience. According to transcriptome sequencing analysis, sHsps family numbers are highly expressed in heat acclimated *M. sieversii* in both their native habitat of Xinjiang and *in vitro*. Previous studies confirmed that sHsps have multiple effects in various abiotic stress and growth processes in numerous plant species. However, it is not clear how many family members are involved in thermal responses in woody plants. Based on RNA-Seq of *M. sieversii* in T3 and T7, we screened 12 sHsps based on Nr annotation and BLAST genomic sequence information in the NCBI database. In all members, 10 candidates have conservative ACD domain and were up-regulated under high temperature conditions, indicating an involvement in the thermal response.

In order to further analyze the functions of *MsHsp16.9*, we cloned the full-length CDS of *MsHsp16.9* and generated *MsHsp16.9*-overexpressing *Arabidopsis* plants. RT-qPCR showed that *MsHsp16.9* was expressed in all tissues, and most strongly expressed in the leaf and pod, both sensitive above-ground tissues that prompt a response to abiotic stresses. It is worth noting that the expression of *MsHsp16.9* was significantly higher in the pod than in others, which may explain why transgenic plants bore pods following heat stress but control plants did not. High expression of *MsHsp16.9* in leaf tissue may indicate a role in the protection of the chlorophyll, and maintaining photosynthesis and normal growth.

The adverse effects of heat stress can be mitigated or eliminated by improved thermal tolerance plants using various genetic engineering and transgenic approaches (Rodríguez et al., 2005). Attempts at engineering heat tolerance by overexpression of sHsps and HSF have been limited compared with attempts to improve tolerance to drought, salt or cold stress. Expression of HSF fusion proteins in *Arabidopsis* produced transgenic plants with high *Hsp* expression and enhanced thermal tolerance (Malik et al., 1999), and sHsps were shown to enhance thermal tolerant in transformed tobacco (Lui and Shono, 1999; Sanmiya et al., 2004). Some successful transgenic cases have also been reported in rice, including improved heat tolerance after fusing *Hsp* genes (Murakami et al., 2004). In this study, we engineered *MsHsp16.9* transgenic lines that showed no significant differences in morphology or growth compared with control plants in normal conditions. Similarly, *sHsp* RNAi and overexpression lines did not show significant differences in vegetative and reproductive growth under optimal conditions in a previous study (McLoughlin et al., 2016). However, transgenic lines displayed enhanced heat tolerance and a higher survival rate than WT plants following heat stress acclimation during seeding, growing and bolting stages. The similar results were also observed in *sHsps* transgenic plants from other species. For instance, *OsHsp18.6* from rice and *ZmHsp16.9* from maize enhanced multifarious stress tolerances in transgenic plants (Sun et al., 2012; Wang et al., 2015). In the present study, transgenic plants did not show any significant increase to NaCl, PEG or low temperature stress conditions (data not shown).

SOD, POD, and CAT constitute an important antioxidative system, and their activities serve as indicators of stress tolerance in plants (Kar and Mishra, 1976). Physiological tests revealed enhanced ROS scavenging ability via antioxidative enzymes under heat stress conditions in transgenic plants compared with controls (Figures 9G–I). In particular, CAT activity was greatly elevated in the TP11 transgenic line. Similar effects have been observed in maize (Sun et al., 2012; Li et al., 2016). SOD and POD activities were also increased in all transgenic lines, which may in part account for the higher survival rate of transgenic plants. Additionally, the observed reduction in MDA in *MsHsp16.9*-overexpressing plants indicates protection of membrane integrity. Furthermore, NBT and DAB staining showed that O_2_- and H_2_O_2_ accumulation was lower in transgenic lines than in control plants under heat stress condition (Figures 11C,D). This also indicates that damage from peroxides was diminished. CAT gene expression was in accordance with CAT activity in TP11 plants (Figure 13). Expression of APX, a scavenger of H_2_O_2_, was also improved in TP3 plants (Figure 13). The above results suggest *MsHsp16.9* may directly or indirectly affect the protective antioxidant enzyme system under heat stress conditions, especially when *MsHsp16.9* is highly expressed.

AtADC1 is involved in polyamine (PA) biosynthetic pathways and responses to abiotic stresses (Sánchez-Rangel et al., 2016). SAMDC expression elevates endogenous spermine levels that impact on both biotic and abiotic stresses (Marco et al., 2014). P5CS is a bifunctional enzyme involved in the accumulation of proline in response to osmotic stress (Pérez-Arellano et al., 2010). Expression of these genes was elevated in transgenic lines (Figure 13), which may influence the activity of cells via the synthesis of secondary metabolites. In ABA-independent regulation, ERD and DREB are rapidly activated during drought stress (Alves et al., 2011; Singh and Laxmi, 2015). Plants under high temperature conditions can lose water and wilt, and ERD and DREB may amplify the signal that connects the relevant response pathways (Figure 13). These results suggest that *MsHsp16.9* may modulate the expression of genes involved in ABA-independent signaling pathways in response to heat stress.

Transgenic expression of the *Trichoderma harzianum* Hsp70 gene in *Arabidopsis* improved resistance to heat and other abiotic stresses, but HSF and four HSP genes were down-regulated in 35S:*Hsp70* plants (Montero-Barrientos et al., 2010). This indicates that *Hsp70* may act as a negative regulator of HSF transcriptional activity in *Arabidopsis* (Montero-Barrientos et al., 2010). To further investigate the performance of *MsHsp16.9*-overexpressing plants under heat stress and have an insight into the molecular mechanisms of *MsHsp16.9*-mediated regulation, we carried out a large-scale screen of HSF gene expression in controls and *MsHsp16.9* transgenic plants. Transgenic plants overexpressing *MsHsp16.9* exhibited significant differences in the expression of HSF regulatory proteins in heat response pathways. Specifically, AtHSFA1D, AtHSFA1E, AtHSFA3, and AtHSFA4A expression in *MsHsp16.9*-overexpressing plants was up-regulated between 3- and 10-fold compared with control plants (Figure 13).

sHsps are important for heat tolerance, since they interact with Hsp101 to protect a set of heat-sensitive proteins involved in protein translation (McLoughlin et al., 2016). Stress-induced and responsive protein members of the Hsp70 family are required for reassembly or depolymerization of misfolded or damaged proteins in plants under stress conditions (Frydman, 2001). *MsHsp16.9* may prevent or reverse inactivation and degradation of heat-sensitive proteins, and then maintain cell equilibrium and steady state that is disrupted by adverse environmental conditions. This inference is in line with the flexible functions of sHsps and α-crystallins (Basha et al., 2012). The sHsps are deemed to be the first defense line by interacting with denatured proteins to prevent their aggregation or present them for ATP-dependent degradation (Haslbeck and Vierling, 2015). In this study, *Hsp70* expression in transgenic lines was up-regulated compared with WT and VC plants, suggesting *MsHsp16.9* may work alongside Hsp70 in the protection of cellular proteins (Figure 13).

## Conclusion

On the whole, we identified 12 sHsps from *M. sieversii* growing in high temperature arid region, along with DEGs from RNA-Seq data. Our findings demonstrated that 10 sHsps were rapidly induced by heat stress, both in the native environment and *in vitro*. Additionally, overexpression of *MsHsp16.9* in *Arabidopsis* provided significant protection against the damaging effects of high temperature stress. Our results suggest that *MsHsp16.9* is a protein chaperone that positively regulates antioxidant enzyme activity and ABA-dependent and independent signaling to attenuate plant responses to adverse environmental stresses, which help to explain the evident prosperity of *M. sieversii* in high temperature environments.

## Author contributors

MY, WS, WY, and GY designed the experiments, analyzed the data and wrote the manuscript. MY and YZ performed the main experiments. HZ and HW contributed in the stress experiments. BH and HL contributed to data analyses and discussion. SC contributed to the production and processing of the pictures. MY, TW, and LZ made a significant contribution for manuscript finishing touches.

### Conflict of interest statement

The authors declare that the research was conducted in the absence of any commercial or financial relationships that could be construed as a potential conflict of interest.

## Supplementary material

Figure S1 T-DNA regions of binary vectors employed for *Agrobacterium tumefaciens*-mediated transformation. **(A)** Diagram of binary vector pCAMBIA3301. **(B)** Binary vector p35S::*MsHsp16.9*.

Figure S2 Heat map showing *MsHsp20* gene expression patterns in *Malus sieversii* under normal (CK) and heat stress conditions. The relative expression levels were calculated using the 2^−ΔΔCT^ method. The heat map was created using Heml 1.0.1.

## References

[B1] Ahmed I. Islam M. Arshad W. Mannan A. Ahmad W. Mirza B. (2009). High-quality plant DNA extraction for PCR: an easy approach. J. Appl. Genet. 50, 105–107. 10.1007/BF03195661 19433907

[B2] Alves M. S. Fontes E. P. B. Fietto L. G. (2011). EARLY RESPONSIVE to DEHYDRATION 15, a new transcription factor that integrates stress signaling pathways. Plant Signal. Behav. 6, 1993–1996. 10.4161/psb.6.12.18268 22105026 PMC3337193

[B3] Apweiler R. Bairoch A. Wu C. H. Barker W. C. Boeckmann B. Ferro S.. (2004). UniProt: the Universal Protein knowledgebase. Nucleic Acids Res. 32 (Suppl. 1), D115–D119. 10.1093/nar/gkh131 14681372 PMC308865

[B4] Ashburner M. Ball C. A. Blake J. A. Botstein D. Butler H. Cherry J. M.. (2000). Gene Ontology: tool for the unification of biology. Nat. Genet. 25, 25–29. 10.1038/75556 10802651 PMC3037419

[B5] Basha E. Friedrich K. L. Vierling E. (2006). The N-terminal arm of small heat shock proteins is important for both chaperone activity and substrate specificity. J. Biol. Chem. 281, 39943–39952. 10.1074/jbc.M607677200 17090542

[B6] Basha E. O'Neill H. Vierling E. (2012). Small heat shock proteins and α-crystallins: dynamic proteins with flexible functions. Trends Biochem. Sci. 37, 106–117. 10.1016/j.tibs.2011.11.005 22177323 PMC3460807

[B7] Clough S. J. Bent A. F. (1998). Floral dip: a simplified method for Agrobacterium-mediated transformation of *Arabidopsis thaliana*. Plant J. 16, 735–743. 10.1046/j.1365-313x.1998.00343.x 10069079

[B8] Cramer G. R. Urano K. Delrot S. Pezzotti M. Shinozaki K. (2011). Effects of abiotic stress on plants: a systems biology perspective. BMC Plant Biol. 11:163. 10.1186/1471-2229-11-163 22094046 PMC3252258

[B9] Dafny-Yelin M. Tzfira T. Vainstein A. Adam Z. (2008). Non-redundant functions of sHSP-CIs in acquired thermotolerance and their role in early seed development in *Arabidopsis*. Plant Mol. Biol. 67, 363–373. 10.1007/s11103-008-9326-4 18379884

[B10] Eylesa S. J. Gierasch L. M. (2010). Nature's molecular sponges: Small heat shock proteins grow into their chaperone roles. Proc. Natl. Acad. Sci. U.S.A. 107, 2727–2728. 10.1073/pnas.0915160107 20133678 PMC2840345

[B11] Finkelstein R. R. Gampala S. S. L. Rock C. D. (2002). Abscisic acid signaling in seeds and seedlings. Plant Cell 14(Suppl. 1), S15–S45. 10.1105/tpc.010441 12045268 PMC151246

[B12] Finn R. D. Bateman A. Clements J. Coggill P. Eberhardt R. Y. Eddy S. R.. (2014). Pfam: the protein families database. Nucleic Acids Res. 42, D222–D230. 10.1093/nar/gkt1223 24288371 PMC3965110

[B13] Frydman J. (2001). Folding of newly translated proteins *in vivo*: the role of molecular chaperones. Annu. Rev. Biochem. 70, 603–647. 10.1146/annurev.biochem.70.1.603 11395418

[B14] Guo M. Liu J.-H. Lu J.-P. Zhai Y.-F. Wang H. Gong Z.-H.. (2015). Genome-wide analysis of the CaHsp20 gene family in pepper: comprehensive sequence and expression profile analysis under heat stress. Front. Plant Sci. 6:806. 10.3389/fpls.2015.00806 26483820 PMC4589653

[B15] Haslbeck M. Vierling E. (2015). A first line of stress defense: small heat shock proteins and their function in protein homeostasis. J. Mol. Biol. 427, 1537–1548. 10.1016/j.jmb.2015.02.002 25681016 PMC4360138

[B16] Himmelbach A. Yang Y. Grill E. (2003). Relay and control of abscisic acid signaling. Curr. Opin. Plant Biol. 6, 470–479. 10.1016/S1369-5266(03)00090-6 12972048

[B17] Hu W. Hu G. Han B. (2009). Genome-wide survey and expression profiling of heat shock proteins and heat shock factors revealed overlapped and stress specific response under abiotic stresses in rice. Plant Sci. 176, 583–590. 10.1016/j.plantsci.2009.01.016 26493149

[B18] Kanehisa M. Goto S. Kawashima S. Okuno Y. Hattori M. (2004). The KEGG resource for deciphering the genome. Nucleic Acids Res. 32(Suppl. 1), D277–D280. 10.1093/nar/gkh063 14681412 PMC308797

[B19] Kar M. Mishra D. (1976). Catalase, peroxidase, and polyphenoloxidase activities during rice leaf senescence. Plant Physiol. 57, 315–319. 10.1104/pp.57.2.315 16659474 PMC542015

[B20] Kaur H. Petla B. P. Kamble N. U. Singh A. Rao V. Salvi P.. (2015). Differentially expressed seed aging responsive heat shock protein OsHSP18.2 implicates in seed vigor, longevity and improves germination and seedling establishment under abiotic stress. Front. Plant Sci. 6:713. 10.3389/fpls.2015.00713 26442027 PMC4568394

[B21] Kim D. H. Xu Z.-Y. Hwang I. (2013). AtHSP17.8 overexpression in transgenic lettuce gives rise to dehydration and salt stress resistance phenotypes through modulation of ABA-mediated signaling. Plant Cell Rep. 32, 1953–1963. 10.1007/s00299-013-1506-2 24081610

[B22] Kirschner M. Winkelhaus S. Thierfelder J. M. Nover L. (2000). Transient expression and heat-stress-induced co-aggregation of endogenous and heterologous small heat-stress protein in tobacoo protoplasts. Plant J. 3, 397–411. 10.1046/j.1365-313x.2000.00887.x 11069712

[B23] Koonin E. V. Fedorova N. D. Jackson J. D. Jacobs A. R. Krylov D. M. Makarova K. S.. (2004). A comprehensive evolutionary classification of proteins encoded in complete eukaryotic genomes. Genome Biol. 5:R7. 10.1186/gb-2004-5-2-r7 14759257 PMC395751

[B24] Kotak S. Larkindale J. Lee U. Koskull-Döring P. V. Vierling E. Scharf K. (2007). Complexity of the heat stress response in plants. Curr. Opin. Plant Biol. 10, 310–316. 10.1016/j.pbi.2007.04.011 17482504

[B25] Leng N. Dawson J. A. Thomson J. A. Ruotti V. Rissman A. I. Smits B. M. G.. (2013). EBSeq: an empirical Bayes hierarchical model for inference in RNA-seq experiments. Bioinformatics 29, 1035–1043. 10.1093/bioinformatics/btt087 23428641 PMC3624807

[B26] Li J. Zhang J. Jia H. Li Y. Xu X. Wang L.. (2016). The Populus trichocarpa PtHSP17.8 involved in heat and salt stress tolerances. Plant Cell Rep. 35, 1587–1599. 10.1007/s00299-016-1973-3 27021382

[B27] Lopes-Caitar V. S. Carvalho M. C. D. Darben L. M. Kuwahara M. K. Nepomuceno A. L. Dias W. P.. (2013). Genome-wide analysis of the Hsp20 gene family in soybean: comprehensive sequence, genomic organization and expression profile analysis under abiotic and biotic stresses. BMC Genomics 14:577. 10.1186/1471-2164-14-577 23985061 PMC3852298

[B28] Lui J. Shono M. (1999). Characterization of mitochondria-located small heat shock protein from Tomato (*Lycopersicon esculentum*). Plant Cell Physiol. 40, 1297–1304. 10.1093/oxfordjournals.pcp.a029518 10682350

[B29] Malik M. K. Slovin J. P. Hwang C. H. Zimmerman J. L. (1999). Modified expression of a carrot small heat shock protein gene, Hsp17.7, results in increased or decreased thermotolerance. Plant J. 20, 89–99. 10.1046/j.1365-313X.1999.00581.x 10571868

[B30] Marco F. Busó E. Carrasco P. (2014). Overexpression of SAMDC1 gene in *Arabidopsis thaliana* increases expression of defense-related genes as well as resistance to *Pseudomonas syringae* and *Hyaloperonospora arabidopsidis*. Front. Plant Sci. 5:115. 10.3389/fpls.2014.00115 24734036 PMC3973925

[B31] McLoughlin F. Basha E. Fowler M. E. Kim M. Bordowitz J. Katiyar-Agarwal S.. (2016). Class I and II small heat-shock proteins protect protein translation factors during heat stress. Plant Physiol. 172, 1221–1236. 10.1104/pp.16.00536 27474115 PMC5047077

[B32] Meisel L. Fonseca B. González S. Baeza-Yates R. Cambiazo V. Campos R.. (2005). A rapid and efficient method for purifying high quality total RNA from Peaches (*Prunus persica*) for functional genomics analyses. Biol. Res. 38, 83–88. 10.4067/S0716-97602005000100010 15977413

[B33] Montero-Barrientos M. Hermosa R. Cardoza R. E. Gutiérrez S. Nicolás C. Monte E. (2010). Transgenic expression of the *Trichoderma harzianum* hsp70 gene increases *Arabidopsis* resistance to heat and other abiotic stresses. J. Plant Physiol. 167, 659–665. 10.1016/j.jplph.2009.11.012 20080316

[B34] Murakami T. Matsuba S. Funatsuki H. Kawaguchi K. Saruyama H. Tanida M. (2004). Over-expression of a small heat shock protein, sHSP17.7, confers both heat tolerance and UV-B resistance to rice plants. Mol. Breed. 13, 165–175. 10.1023/B:MOLB.0000018764.30795.c1

[B35] Ouyang Y. Chen J. Xie W. Wang L. Zhang Q. (2009). Comprehensive sequence and expression profile analysis of Hsp20 gene family in rice. Plant Mol. Biol. 70, 341–357. 10.1007/s11103-009-9477-y 19277876

[B36] Pérez-Arellano I. Carmona-Álvarez F. Martínez A. I. Rodríguez-Díaz J. Cervera J. (2010). Pyrroline-5-carboxylate synthase and proline biosynthesis: From osmotolerance to rare metabolic disease. Protein Sci. 19, 372–382. 10.1002/pro.340 20091669 PMC2866264

[B37] Ré M. D. Gonzalez C. Escobar M. R. Sossi M. L. Valle E. M. Boggio S. B. (2017). Small heat shock proteins and the postharvest chilling tolerance of tomato fruit. Physiol. Plant. 159, 148–160. 10.1111/ppl.12491 27545651

[B38] Rodríguez M. Canales E. Borras-Hidalgo O. (2005). Molecular aspects of abiotic stress in plants. Biotecnol. Aplicada 22, 1–10. Available online at: https://www.researchgate.net/publication/237502514_Molecular_aspects_of_abiotic_stress_in_plants

[B39] Roychoudhury A. Paul S. Basu S. (2013). Cross-talk between abscisic acid-dependent and abscisic acid-independent pathways during abiotic stress. Plant Cell Rep. 32, 985–1006. 10.1007/s00299-013-1414-5 23508256

[B40] Ruibal C. Castro A. Carballo V. Szabados L. Vidal S. (2013). Recovery from heat, salt and osmotic stress in *Physcomitrella patens* requires a functional small heat shock protein PpHsp16.4. BMC Plant Biol. 13:174. 10.1186/1471-2229-13-174 24188413 PMC4228350

[B41] Sánchez-Rangel D. Chávez-Martínez A. I. Rodríguez-Hernández A. A. Maruri-López I. Urano K. Shinozaki K.. (2016). Simultaneous silencing of two arginine decarboxylase genes alters development in *Arabidopsis*. Front. Plant Sci. 7:300. 10.3389/fpls.2016.00300 27014322 PMC4789552

[B42] Sanmiya K. Suzuki K. Egawa Y. Shono M. (2004). Mitochondrial small heat-shock protein enhances thermotolerance in tobacco plants. FEBS Lett. 557:265–268. 10.1016/S0014-5793(03)01494-7 14741379

[B43] Scharf K.-D. Siddique M. Vierling E. (2001). The expanding family of *Arabidopsis thaliana* small heat stress proteins and a new family of proteins containing α-crystallin domains (Acd proteins). Cell Stress Chaperones 6, 225–237. 10.1379/1466-1268(2001)006<0225:TEFOAT>2.0.CO;2 11599564 PMC434404

[B44] Shi J. Fu X.-Z. Peng T. Huang X.-S. Fan Q.-J. Liu J.-H. (2010). Spermine pretreatment confers dehydration tolerance of citrus *in vitro* plants via modulation of antioxidative capacity and stomatal response. Tree Physiol. 30, 914–922. 10.1093/treephys/tpq030 20462936

[B45] Singh D. Laxmi A. (2015). Transcriptional regulation of drought response: a tortuous network of transcriptional factors. Front. Plant Sci. 6:895. 10.3389/fpls.2015.00895 26579147 PMC4625044

[B46] Sun L. Liu Y. Kong X. Zhang D. Pan J. Zhou Y.. (2012). ZmHSP16.9, a cytosolic class I small heat shock protein in maize (*Zea mays*), confers heat tolerance in transgenic tobacco. Plant Cell Rep. 31, 1473–1484. 10.1007/s00299-012-1262-8 22534681

[B47] Sun W. Van Montagu M. Verbruggen N. (2002). Small heat shock proteins and stress tolerance in plants. Biochim. Biophys. Acta 1577, 1–9. 10.1016/S0167-4781(02)00417-7 12151089

[B48] Sun X. Sun C. Li Z. Hu Q. Han L. Luo H. (2016). AsHSP17, a creeping bentgrass small heat shock protein modulates plant photosynthesis and ABA-dependent and independent signalling to attenuate plant response to abiotic stress. Plant Cell Environ. 39, 1320–1337. 10.1111/pce.12683 26610288

[B49] Tatusov R. L. Galperin M. Y. Natale D. A. Koonin E. V. (2000). The GOG database: a tool for genome-scale analysis of protein functions and evolution. Nucleic Acids Res. 28, 33–36. 10.1093/nar/28.1.33 10592175 PMC102395

[B50] Wang A. Yu X. Mao Y. Liu Y. Liu G. Liu Y. (2015). Overexpression of a small heat-shock-protein gene enhances tolerance to abiotic stresses in rice. Plant Breed. 134, 384–393. 10.1111/pbr.12289

[B51] Waters E. R. Aevermann B. D. Sanders-Reed Z. (2008). Comparative analysis of the small heat shock proteins in three angiosperm genomes identifies new subfamilies and reveals diverse evolutionary patterns. Cell Stress Chaperones 13, 127–142. 10.1007/s12192-008-0023-7 18759000 PMC2673885

[B52] Wei T. Deng K. Gao Y. Liu Y. Yang M. Zhang L.. (2016). *Arabidopsis* DREB1B in transgenic Salvia miltiorrhiza increased tolerance to drought stress without stunting growth. Plant Physiol. Biochem. 104, 17–28. 10.1016/j.plaphy.2016.03.003 27002402

[B53] Xiong L. Schumaker K. S. Zhu J.-K. (2002). Cell signaling during cold, drought, and salt stress. Plant Cell 14(Suppl. 1), S165–S183. 12045276 10.1105/tpc.000596 PMC151254

[B54] Yan G. Long H. Song W. Chen R. (2008). Genetic polymorphism of *Malus sieversii* populations in Xinjiang, China. Genet. Resour. Crop Evol. 55, 171–181. 10.1007/s10722-007-9226-5

[B55] Yu X. Wang H. Lu Y. de Ruiter M. Cariaso M. Prins M.. (2012). Identification of conserved and novel microRNAs that are responsive to heat stress in *Brassica rapa*. J. Exp. Bot. 63, 1025–1038. 10.1093/jxb/err337 22025521 PMC3254694

[B56] Zhang J. Li J. Liu B. Zhang L. Chen J. Lu M. (2013). Genome-wide analysis of the Populus Hsp90 gene family reveals differential expression patterns, localization, and heat stress responses. BMC Genomics 14:532. 10.1186/1471-2164-14-532 23915275 PMC3750472

[B57] Zhang L. Zhang Q. Gao Y. Pan H. Shi S. Wang Y. (2014). Overexpression of heat shock protein gene PfHSP21.4 in *Arabidopsis thaliana* enhances heat tolerance. Acta Physiol. Plantarum 36, 1555–1564. 10.1007/s11738-014-1531-y

