# Supplementary figures and images for: Identification of MsHsp20 Gene Family in Malus sieversii and Functional Characterization of MsHsp16.9 in Heat Tolerance
Source: Front Plant Sci. 2017 Nov 1;8:1761. doi: 10.3389/fpls.2017.01761 (PMC5672332; doi:10.3389/fpls.2017.01761)

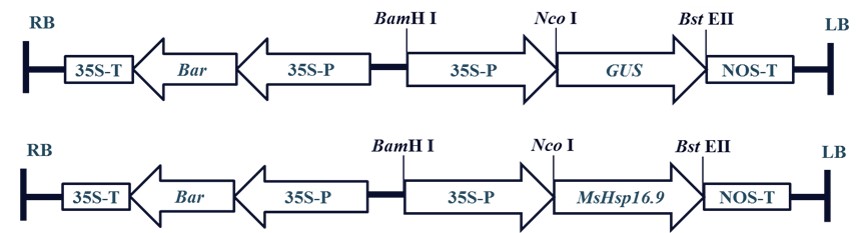

Supplement: Figure S1 — T-DNA regions of binary vectors employed for Agrobacterium tumefaciens-mediated transformation. (A) Diagram of binary vector pCAMBIA3301. (B) Binary vector p35S::MsHsp16.9. [file Image1.JPEG]

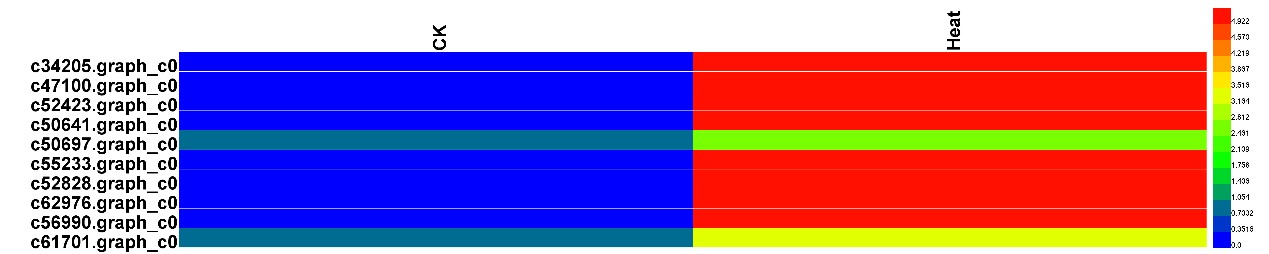

Supplement: Figure S2 — Heat map showing MsHsp20 gene expression patterns in Malus sieversii under normal (CK) and heat stress conditions. The relative expression levels were calculated using the 2−ΔΔCT method. The heat map was created using Heml 1.0.1. [file Image2.JPEG]
