# Supplementary material for: Identification of MsHsp20 Gene Family in Malus sieversii and Functional Characterization of MsHsp16.9 in Heat Tolerance
Source: Front Plant Sci. 2017 Nov 1;8:1761. doi: 10.3389/fpls.2017.01761 (PMC5672332; doi:10.3389/fpls.2017.01761)
Supplement: Supplementary file 3 [file Table1.DOCX]

**Table S1 The location and weather characters of two materials (T3 and T7)**

| Materials number | Location | Altitude (m) | Sampling day temperature  (℃) | Annual evaporation(mm) | The annual average temperature (℃) | ≥10℃accumulated temperature  (℃) |
| --- | --- | --- | --- | --- | --- | --- |
| T3 | N 44°25'39"  E 80°47'18" | 1180 | 25 | 1887 | 9.0 | 3503 |
| T7 | N 43°23'15"  E 83°34'57" | 1340 | 18 | 1285 | 8.1 | 2952 |
