# Supplementary material for: Identification of MsHsp20 Gene Family in Malus sieversii and Functional Characterization of MsHsp16.9 in Heat Tolerance
Source: Front Plant Sci. 2017 Nov 1;8:1761. doi: 10.3389/fpls.2017.01761 (PMC5672332; doi:10.3389/fpls.2017.01761)
Supplement: Supplementary file 4 [file Table2.DOCX]

**Table S2 Oligos used in this study**

| Oligo name and sequence **(5’ to 3’)** for RNA-Seq verification | | | |
| --- | --- | --- | --- |
| c57752.graph_c0-F  c57752.graph_c0-R | AATGGTTTGGAACTGGAGGACAAG  GAGGAAGAAACCGAGCATGGAAAA | c50011.graph_c0-F  c50011.graph_c0-R | TTCCAAAACCCAATCC  TTGAATACGAAGCTCACTG |
| c30710.graph_c0-F  c30710.graph_c0-R | AGTATCCAGGGAGTTCAGGTGCAT  GAGCTGCAACATCCTCAGTCAGTT | c51187.graph_c0-F  c51187.graph_c0-R | CGCATTTCAGTTTTCAA;  AAAATACCCCTAGACTTTGTA |
| c62181.graph_c0 c62181.graph_c0 | CCTTTCCTCTCTAACGTACACATC;  TCCTCCTGTCAATAGAAATATCCT | c60022.graph_c0-F  c60022.graph_c0-R | ATCTGGAAGCCACAGACT;  TTGACGCTTGATGACACT |
| c50751.graph_c0-F  c50751.graph_c0-R | CTCTCTTAAAAGCAAACATGGCTG;R:AAATGGTAGAAATGAGGCTAATGA | c54967.graph_c0-F  c54967.graph_c0-R | AGAAACTTAACATATCCAAAAACC;R:TCTCTTAATACCATGAAACAACTA |
| c61654.graph_c0-F  c61654.graph_c0-R | TTGGTCTGTTACGGTCAT;R:AGGTGTGGTGTGTTAAAAGT | c45514.graph_c0-F  c45514.graph_c0-R | CTGCTCTGAGTCACGTTGTTGCTTT;R:CCTATATGCCTTGAATCTCCATCGC |
| c42290.graph_c0-F  c42290.graph_c0-R | GGCTGGGGATTTTGGGTTTGA;  CAAATGAGTCCACAGCGAGAAACAG | c44413.graph_c0-F  c44413.graph_c0-R | GCCTCAGACCCAAAATCTCATTCTC;  AAACAGCATTAGGCTTGTTGTGGTC |
| c47100.graph_c0-F  c47100.graph_c0-R | GATGATGTTGTTAGGCCTCATGGCC;GCACCCTGTTCTCCTCCACCTCTAT | c61824.graph_c0-F  c61824.graph_c0-R | TGTGAGAGGGCAAAGAGGACTCTCT;R:GTACCTTCGGAATCCTAGTAGAGCC |
| c60991.graph_c0-F  c60991.graph_c0-R | GTTCTTGCAGAGAATGAGAGCCAGA;R:TGTTTTTCCTGATGAAGGAGGTCCC | c43838.graph_c0-F  c43838.graph_c0-R | CAAATGAGTCCACAGCGAGAAACAG;  GCAGAACAGGATAACCCCCATAGAA |
| c49545.graph_c0-F  c49545.graph_c0-R | GTCATTCCCCCACCTAGATTGTTC;R:CTTGTCAACCCCATCGATTCTGT | c52530.graph_c0-F  c52530.graph_c0-R | TCTGTGTTGCAAACTACAAAGCTCC;  GAGCAACAAGTAGGATTTCCGAACC |
| Oligo name and sequence (**5’ to 3’**) for construction of *MsHsp16.9* and positive identification | | | |
| MsHSP16.9-F  MsHSP16.9-R | ACCATGGATGTCGCTGATTCC  GCGGTTACCTCAACCAGAGATCT | 35S-F  35S-R | AACAGAACTCGCCGTAAAG TAGTGGGATTGTGCGTCAT |
| MsActin-F  MsActin-R | TACTGCTGAGCGGGAAATTGTG GCTCCGATAGTGATTACCTGTCCAT | AtActin-F  AtActin-R | CCATGGAGAATGTGGGATCTGAATCATATGC  GGTCACCCTCTCAACCTAGAGACAAATTCAATG |
| Oligo name and sequence (**5’ to 3’**) for MssHsp expression at 25°C (normal) and 42°C (heat stress) | | | |
| c34205.graph_c0-F  c34205.graph_c0-R | AGAACATAAACAGGCTGCTTGAC  AATACTCATCATCCGACCCTTCC | c47100.graph_c0-F  c47100.graph_c0-R | CAAACCAATGCCTTGATTCCGTAC  GCACCCTGTTCTCCTCCACCTCT |
| c52423.graph_c0-F  c52423.graph_c0-R | ATCCAATGGGTCCTTGCTGC  CCGGTTCTCCTCAACCTCGA | c50641.graph_c0-F  c50641.graph_c0-R | ACCAACCCAACTTTGCTCCTTTC  GCTCCAGCTTCACGTCCTCTTTT |
| c50697.graph_c0-F  c50697.graph_c0-R | TCCTTACCAATCTGTCTTCCCTT CACTGAGTCCTCCACTTCTACTTTT | c55233.graph_c0-F  c55233.graph_c0-R | TTTCTGGCAGGGCTACAGGG  CCTTCGCCTTCGGAATCACC |
| c52828.graph_c0-F  c52828.graph_c0-R | CATTCAACATGGTCGTCGGTTAG  GTATTGCTACAAGGGCATTCAGG | c62976.graph_c0-F  c62976.graph_c0-R | TTGGTGACCTCAATGACCGACTG  CCTAATGAACTTGCCACTGCTCC |
| c56990.graph_c0-F  c56990.graph_c0-R | CCCTCAATCTGTGGGACCCTTTC  TCGTCTTCCACCTCCACCTTCAC | c61701.graph_c0-F  c61701.graph_c0-R | CCCACGCGCACATCGACT  GAGAGGAAGAGGGAGCAGGAGG |
