# Supplementary material for: Identification of MsHsp20 Gene Family in Malus sieversii and Functional Characterization of MsHsp16.9 in Heat Tolerance
Source: Front Plant Sci. 2017 Nov 1;8:1761. doi: 10.3389/fpls.2017.01761 (PMC5672332; doi:10.3389/fpls.2017.01761)
Supplement: Supplementary file 5 [file Table3.DOCX]

**Table S3 Gene IDs of *Hsp20* members and phylogenetic classification**

| Gene Name | Gene ID |
| --- | --- |
| Malus x domestica 16.9 kDa class I heat shock protein 2 | LOC103431125 |
| Pyrus x bretschneideri 16.9 kDa class I heat shock protein 2 | LOC103942692 |
| Malus x domestica 18.8 kDa class II heat shock protein | LOC103440887 |
| Malus x domestica 18.5 kDa class I heat shock protein | LOC103447478 |
| Malus x domestica 17.8 kDa class I heat shock protein | LOC103406609 |
| Prunus mume 18.5 kDa class I heat shock protein | LOC103320130 |
| Malus x domestica 18.1 kDa class I heat shock protein | LOC103409029 |
| Pyrus x bretschneideri 18.1 kDa class I heat shock protein | LOC103953301 |
| Malus x domestica 15.4 kDa class V heat shock protein | LOC103446712 |
| Pyrus x bretschneideri 15.4 kDa class V heat shock protein | LOC103948158 |
| Pyrus x bretschneideri 17.1 kDa class II heat shock protein | LOC103927499 |
| Malus x domestica 17.4 kDa class III heat shock protein | LOC103454275 |
| Malus x domestica 22.0 kDa class IV heat shock protein | LOC103435785 |
| Pyrus x bretschneideri 22.0 kDa class IV heat shock protein | LOC103960307 |
| Malus x domestica 15.7 kDa heat shock protein | LOC103445871 |
| Pyrus x bretschneideri 15.7 kDa heat shock protein | LOC103959434 |
| Malus x domestica 21.7 kDa class VI heat shock protein | LOC103448540 |
| Malus x domestica 26.5 kDa heat shock protein, mitochondrial-like | LOC103428235 |
| Pyrus x bretschneideri 26.5 kDa heat shock protein, mitochondrial | LOC103955267 |
| Malus x domestica 17.1 kDa class II heat shock protein-like | LOC103410643 |
| Malus x domestica 23.5 kDa heat shock protein, mitochondrial-like | LOC103403249 |
| Malus x domestica 23.6 kDa heat shock protein, mitochondrial-like | LOC103439629 |
| Malus x domestica cultivar Fuji heat shock protein 17.5 | EU636239 |
