# Supplementary material for: Identification of MsHsp20 Gene Family in Malus sieversii and Functional Characterization of MsHsp16.9 in Heat Tolerance
Source: Front Plant Sci. 2017 Nov 1;8:1761. doi: 10.3389/fpls.2017.01761 (PMC5672332; doi:10.3389/fpls.2017.01761)
Supplement: Supplementary file 6 [file Table4.DOCX]

**Table S4 Data evaluation of RNA-Seq of T7 and T3 *Malus sieversii***

| Samples | Clean reads | GC Content | %≥Q30 |
| --- | --- | --- | --- |
| T7 | 25,641,037 | 47.64% | 85.01% |
| T3 | 28,821,560 | 47.55% | 85.05% |
