# Supplementary material for: Identification of MsHsp20 Gene Family in Malus sieversii and Functional Characterization of MsHsp16.9 in Heat Tolerance
Source: Front Plant Sci. 2017 Nov 1;8:1761. doi: 10.3389/fpls.2017.01761 (PMC5672332; doi:10.3389/fpls.2017.01761)
Supplement: Supplementary file 7 [file Table5.DOCX]

**Table S5 The assembly results of RNA-Seq of T7 and T3 *Malus sieversii***

| Length Range | Contig | Transcript | Unigene |
| --- | --- | --- | --- |
| 200-300 | 2,456,121(97.54%) | 26,013(12.56%) | 20,105(31.96%) |
| 300-500 | 27,717(1.10%) | 26,449(12.77%) | 16,413(26.09%) |
| 500-1000 | 17,758(0.71%) | 35,533(17.16%) | 11.355(18.05%) |
| 1000-2000 | 10.449(0.41%) | 58.211(28.11%) | 7,880(12.53%) |
| 2000+ | 6,148(0.24%) | 60,878(29.40%) | 7,159(11.38%) |
| Total Number | 2,518,193 | 207,084 | 62,912 |
