# Supplementary material for: Identification of MsHsp20 Gene Family in Malus sieversii and Functional Characterization of MsHsp16.9 in Heat Tolerance
Source: Front Plant Sci. 2017 Nov 1;8:1761. doi: 10.3389/fpls.2017.01761 (PMC5672332; doi:10.3389/fpls.2017.01761)
Supplement: Supplementary file 8 [file Table6.DOCX]

**Table S6 Heat stress response-related *MsHsp20* genes in *Malus Sieversii***

| Biomarker #ID | T07 | T03 | FDR | log_2_FC | Up/down-regulated | Nr_annotation |
| --- | --- | --- | --- | --- | --- | --- |
| c34205.graph_c0 | 7.594597 | 306.7043 | 0 | 4.867473 | up | 26.5 kDa heat shock protein |
| c47100.graph_c0 | 4.707918 | 624.8559 | 0 | 6.574103 | up | 22.7 kDa class IV heat shock protein |
| c52423.graph_c0 | 0.771029 | 307.6577 | 0 | 8.043193 | up | 22.0 kDa class IV heat shock protein |
| c30087.graph_c0 | 39.59863 | 352.1403 | 5.29E-07 | 2.694845 | up | 15.7 kDa heat shock protein |
| c50641.graph_c0 | 3.492258 | 18.49405 | 0.002321 | 1.918556 | up | 17.5 kDa class I heat shock protein |
| c33770.graph_c0 | 1.638066 | 121.5404 | 0 | 5.665319 | up | 18.8 kDa class II heat shock protein |
| c50697.graph_c0 | 3.13104 | 25.97049 | 4.98E-11 | 3.143572 | up | 15.4 kDa class V heat shock protein |
| c55233.graph_c0 | 176.9865 | 589.3526 | 0.000321 | 1.872196 | up | 23.6 kDa heat shock protein |
| c52828.graph_c0 | 3.218992 | 20.64415 | 1.36E-08 | 2.773183 | up | 17.8 kDa class I heat shock protein |
| c62976.graph_c0 | 1.110854 | 6.699394 | 9.47E-06 | 2.60885 | up | 18.1 kDa class I heat shock protein |
| c56990.graph_c0 | 629.3274 | 1990.761 | 0.001154 | 1.79835 | up | 18.5 kDa class I heat shock protein |
| c61701.graph_c0 | 126.5072 | 6635.134 | 0 | 5.784799 | up | 16.9 kDa class I heat shock protein |
